# *Limosilactobacillus reuteri* promotes the expression and secretion of enteroendocrine‐ and enterocyte‐derived hormones

**DOI:** 10.1096/fj.202401669R

**Published:** 2025-03-18

**Authors:** Sara C. Di Rienzi, Heather A. Danhof, Micah D. Forshee, Ari Roberts, Robert A. Britton

**Affiliations:** ^1^ Department of Molecular Virology and Microbiology Baylor College of Medicine Houston Texas USA; ^2^ Alkek Center for Metagenomics and Microbiome Research Baylor College of Medicine Houston Texas USA

**Keywords:** adipolin, enterocyte, enteroendocrine, GIP, hormone, kisspeptin, *Lactobacillus*, luteinizing hormone, PYY, small intestine, vasopressin

## Abstract

Intestinal microbes can beneficially impact host physiology, prompting investigations into the therapeutic usage of such microbes in a range of diseases. For example, human intestinal microbe *Limosilactobacillus reuteri* strains ATCC PTA 6475 and DSM 17938 are being considered for use for intestinal ailments, including colic, infection, and inflammation, as well as for non‐intestinal ailments, including osteoporosis, wound healing, and autism spectrum disorder. While many of their beneficial properties are attributed to suppressing inflammatory responses, we postulated that *L. reuteri* may also regulate intestinal hormones to affect physiology within and outside of the gut. To determine if *L. reuteri* secreted factors impact the secretion of enteric hormones, we treated an engineered jejunal organoid line, *NGN3*‐HIO, which can be induced to be enriched in enteroendocrine cells, with *L. reuteri* 6475 or 17938 conditioned medium and performed transcriptomics. Our data suggest that these *L. reuteri* strains affect the transcription of many gut hormones, including vasopressin and luteinizing hormone subunit beta, which have not been previously recognized as produced in the gut epithelium. Moreover, we find that these hormones appear to be produced in enterocytes, in contrast to canonical gut hormones produced in enteroendocrine cells. Finally, we show that *L. reuteri* conditioned media promote the secretion of enteric hormones, including serotonin, GIP, PYY, vasopressin, and luteinizing hormone subunit beta, and identify by metabolomics metabolites potentially mediating these effects on hormones. These results support *L. reuteri* affecting host physiology through intestinal hormone secretion, thereby expanding our understanding of the mechanistic actions of this microbe.

## Graphical abstract

The probiotic *Limosilactobacillus reuteri* affects the epithelium of the human small intestine by transcriptionally upregulating and promoting the secretion of intestinal hormones. The hormones that *L. reuteri* regulates include those produced in enteroendocrine cells, like serotonin, GIP, and CCK, and those made in enterocytes, oxytocin, vasopressin, and luteinizing hormone subunit beta.

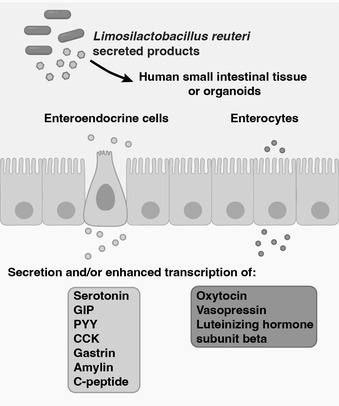

**Abbreviations**
DEG: differentially expressed gene
HIO: human intestinal organoid

## 1 INTRODUCTION

The use of commensal microbes in the treatment of disease has the potential to herald a new era of microbial‐based therapeutics. The human‐associated *Limosilactobacillus reuteri* is one such microbe considered for development as a therapeutic: it has been shown to improve symptoms of infant colic,^1^ osteoporosis,^2^ and inflammatory diseases^3^, ^4^, ^5^, ^6^ and is being considered for its role in alleviating asocial behavior associated with autism spectrum disorder.^7^, ^8^, ^9^, ^10^, ^11^ How *L. reuteri* mediates these effects is not fully understood. Moreover, several different *L. reuteri* strains are currently in use, highlighting the importance of studying strain variation to understand therapeutic efficacy.

Two of the commonly employed strains that are currently marketed as probiotics are *L. reuteri* ATCC PTA 6475 and *L. reuteri* DSM 17938. While both were originally derived from human breast milk, these strains are phylogenetically and functionally distinct. *L. reuteri* 6475 belongs to *L. reuteri* clade II, while *L. reuteri* 17938 (derived from strain ATCC 55730^12^) belongs to *L. reuteri* clade VI.^13^ *L. reuteri* 17938 (or its parent *L. reuteri* 55730) has been demonstrated to reduce infant colic,^1^ assist with feeding tolerance in preterm infants,^14^ improve intestinal motility in preterm^14^ and term infants,^15^ and improve cytokine ratios in children with apoptotic dermatitis.^16^ *L. reuteri* 6475 has been shown to have potential in relieving inflammatory conditions through TNF suppression, which may be linked to its capacity to reduce osteoporosis.^2^, ^17^, ^18^, ^19^, ^20^, ^21^, ^22^, ^23^ *L. reuteri* 6475 has also been demonstrated to be efficacious in promoting wound healing,^24^, ^25^ restoring normal social behavior in mouse models of autism^7^, ^8^, ^9^, ^11^ (which *L. reuteri* 17938 has been shown unable to do so in mice), and improving male reproductive health in mice.^26^ These two strains are similar in their ability to produce the antimicrobial reuterin and the vitamins pseudo B12 and B9 (folate)^27^ and to produce proteins for host mucus adherence.^28^ *L. reuteri* 6475 can also produce histamine, while *L. reuteri* 17938 cannot.^13^ This histamine production is implicated in *L. reuteri* 6475's suppression of the inflammatory signal tumor necrosis factor (TNF).^27^ *L. reuteri* 17938 has also been demonstrated to liberate adenosine from AMP, which may be involved in its function in reducing autoimmunity in Treg deficiency disorders by enhancing CD73^+^CD8^+^T cells.^29^

While many of *L. reuteri*'s functions are thought to be due to interactions with immune cells, *L. reuteri* itself or its secreted products has the capacity to influence host physiology through a wide range of cell types. Particularly in the small intestine, where the mucus layer is thin, *L. reuteri* may have ample opportunities to interact with the host epithelial cells. The diverse roles of *L. reuteri* in gut motility, inflammatory processes, and the gut‐brain axis led us to consider whether some of *L. reuteri*'s interactions with the host are mediated through enteroendocrine cells.

Enteroendocrine cells are secretory cells in the intestine specialized for the secretion of hormones. Enteroendocrine cells sense nutrients like sugars, peptides, and fatty acids in the intestinal lumen through G‐protein coupled receptors and utilize ion (sodium, hydrogen, calcium) transporters to bring nutrients into the cell.^30^ On apical entry or basolateral exit from enteroendocrine cells, these nutrients can trigger hormone receptors and lead to the release of hormones from the apical or basolateral side of the cell.^31^ Enteroendocrine cells also respond to microbial stimulus through toll‐like receptors to release cytokines, subsequently affecting inflammatory responses.^30^ As well, released gut hormones can directly and indirectly influence pro‐ and anti‐inflammatory immune cell populations through a variety of mechanisms.^30^ Finally, enteroendocrine cells and a few specific hormones are associated with the integrity of the intestinal barrier.^30^

Enteroendocrine cells, however, comprise ~1% of gut epithelial cells, thereby making the study of these cells difficult in vivo and in non‐transformed tissue lines. To overcome this limitation, we recently developed a human enteroendocrine‐enriched jejunal organoid line.^32^ Through the induction of the developmental regulator of enteroendocrine cells, *NGN3*, we can increase the number of enteroendocrine cells to ~40% in this adult stem cell‐derived human jejunal organoid line at the expense of enterocytes.^32^

Here, we utilized these *NGN3* human intestinal organoids (HIOs) to characterize how *L. reuteri* secreted products impact enteroendocrine cells. By performing RNA‐Seq on uninduced organoids and induced, enteroendocrine‐enriched organoids, we observe that *L. reuteri* affects the transcription of genes involved in hormone secretion, nutrient sensing, cell adhesion, mucus production, immune/stress response, and cell fate. Among the impacted hormones are enterocyte‐derived hormones not previously characterized in the intestinal epithelium. For several of the impacted hormones, we additionally demonstrate that *L. reuteri* promotes the secretion of these hormones from HIOs or from ex vivo human intestinal tissue. In general, we observe similar effects of *L. reuteri* strains 6475 and 17938 on epithelial cells, but with *L. reuteri* 6475 having a greater magnitude of effect on transcription. Metabolome analysis of the conditioned media produced by these strains suggests that *L. reuteri* 6475 may have a greater impact on gene expression due to the production of more differential metabolites than *L. reuteri* 17938. Collectively, these results suggest specific mechanisms by which *L. reuteri* mediates its beneficial effects with a magnified look at how *L. reuteri* interacts with enteric hormones.

## 2 METHODS

### 2.1 Preparation of bacterial conditioned media

*L. reuteri* strains ATCC PTA 6475 and DSM 17938 were provided by BioGaia (Sweden). A single *L. reuteri* 6475 or 17938 colony from an MRS agar plate was inoculated into 10 mL MRS broth and incubated in a tightly closed conical tube in a 37°C water bath or incubator. After 15 h of incubation, the *L. reuteri* culture was diluted to an OD_600_ of 0.1 into 25 to 40 mL of pre‐warmed LDM4^6^ and placed into a 37°C water bath to incubate until reaching an OD_600_ of 0.5–0.6, at which point the cells are in log phase and have a density of 10^7^–10^8^ cfu/mL. Next, cells were pelleted by centrifugation, and the resulting supernatant was transferred to a new conical tube. The pH of the supernatant was measured by applying 2 μL of the supernatant onto pH paper (range 6.0–8.0, Fisherbrand, Pittsburgh, PA, USA) and adjusted to 7.0 using 10 M sodium hydroxide solution. Neutralized conditioned media and LDM4 media control were filter sterilized (0.22 μm PVDF membrane, Steriflip, EMD Millipore, Burlington, MA, USA), aliquoted, frozen at −80°C overnight, and then lyophilized. Lyophilized conditioned media were stored at −20°C until use.

### 2.2 Propagation of organoids and organoid media

J2 *NGN3* organoids were propagated in 3D in CMGF+ media^32^ + 10 μmol Y‐27632 Rock inhibitor + 200 μg/mL geneticin as previously described.^33^ *NGN3*‐HIOs were then seeded onto 24‐well transwells and differentiated in the presence of differentiation media^32^ with (induced) or without (uninduced) 1 μg/mL doxycycline.

### 2.3 Transwell assay

For use on organoids, *L. reuteri* lyophilized conditioned media were resuspended in an equal volume of organoid differentiation media. The existing differentiation media on the apical side of the transwells were removed and replaced with 100 μL differentiation media supplemented with *L. reuteri* lyophilized conditioned media or lyophilized media control (LDM4). Transwells were incubated for 3 h at 37°C with 5% CO_2_. Following this, apical and basolateral supernatants were removed and stored at −20°C in a 96‐well plate to be used later in a hormone secretion assay. The transwell membrane was removed from the support surface and placed in TRIzol solution (Invitrogen, Waltham, MA, USA). Following a chloroform extraction, the aqueous phase containing total RNA was immediately extracted using a Qiagen RNeasy kit (Qiagen, Germantown, MD, USA). In pilot experiments, the viability of the *NGN3*‐HIOs after *L. reuteri* treatment was checked visually and by performing a PrestoBlue assay (ThermoFisher Scientific, USA).

### 2.4 RNA‐Seq

Paired‐end Illumina sequencing libraries were prepared by Novogene (Sacramento, CA, USA). Briefly, total RNA was enriched for eukaryotic mRNA. mRNA was fragmented to an average insert size of 250 to 300 bp, and cDNA was prepared using the standard NEB library construction method. The library was 150 bp paired‐end sequenced on a NovaSeq 6000. Basecalling was performed using CASAVA v1.8.^34^ Reads were filtered as follows: reads containing adaptors were removed, reads with more than 10% N reads were removed, and reads with >50% of the bases having a Qscore ≤5 were removed.

Sequenced reads were aligned to the human genome hg19 using Star (v2.5)^35^ using the maximal mappable prefix for junction reads and with mismatch = 2. Read counts per gene were tabulated with HTSeq v0.6.1.^36^ The gene count table provided by Novogene was further processed using a pipeline derived from iDEP version 0.82.^37^ Genes were filtered to keep those with at least 1 count per million in 5 samples, thereby retaining 15 369 genes.

For multidimensional scaling, rlog‐transformed data were visualized using a *t*‐distribution to estimate the hypothetical spread of the data. The contribution of induction and *L. reuteri* treatment to the variation in data were modeled using a permutational multivariate analysis of variance (PERMANOVA) of the form: Euclidean distance matrix ~ induction + treatment + induction * treatment using the adonis function in vegan (v2.5–5).^38^

For correlation analyses, rlog‐transformed values were used. Lowly expressed genes belonging to the bottom quartile were removed. Correlations among samples were computed using a Pearson correlation. Correlations were visualized using the ComplexHeatmap package (v2.3.1),^39^ with rows and columns clustered by a Euclidean distance metric and using complete linkage clustering for both. Within and between sample distances were plotted using the ggboxplot function in ggpubr (v0.2.4).^40^ Significance among distances was calculated by a t‐test with a multiple testing correction using Holm's method.^41^ Differences between means (circle size) and adjusted *p*‐values (circle color) were visualized as a correlogram using the ComplexHeatmap package (v2.3.1).^39^

For identification of differentially expressed genes, gene counts were modeled as genecount ~ treatment‐induction + organoid_batch in DESeq2^42^ V1.22.2 using a Wald test with *p*‐values corrected using the Benjamini–Hochberg procedure^43^ with an FDR cutoff of 0.1 and a fold change cutoff of 2. DESeq2 models the underlying variation using a negative binomial distribution. LDM4 (media alone) and uninduced (not enteroendocrine enriched) were used as reference levels.

### 2.5 Functional analyses

Ensembl IDs release 95 were converted to Ensembl IDs release 98 before analyzing for statistical enrichment of gene functions using the Ensembl ID converter.^44^ Annotations for PANTHER GO‐Slim Biological Process, PANTHER GO‐Slim Molecular Function, PANTHER GO‐Slim Cellular Component, PANTHER Protein Class, Panther Pathways, and Reactome^45^, ^46^ were performed in PANTHER,^47^ using a binomial test and a false discovery cutoff of 0.05. Genes belonging to enriched (not depleted) functional categories defined by PANTHER^47^ were searched in GeneCards^48^ and annotated into one of the following broad groups: Cell fate/growth, hormone secretion, immune response, membrane component, mucus, nutrient metabolism/response, signaling, or metal/stress response. Enrichments of these groups within K‐means determined clusters (see below for heatmap visualization) were determined using a hypergeometric distribution, and all *p*‐values across groups and clusters were corrected *en masse* using the Benjamini‐Hochberg^43^ method, whereby FDR values less than 0.1 were considered significant.

### 2.6 Visualization of RNA‐Seq data

For multidimensional scaling, clustering, and heatmap visualization, read counts were transformed using the rlog function from DESeq2 V1.22.2.^42^ For multidimensional scaling analysis, a Euclidean distance metric on the rlog‐transformed data was used. For displaying the gene expression data as a heatmap, the rlog transformed data were batch corrected using the removeBatchEffect command in the limma package,^49^ and the data were centered and scaled using the scale function in base R.^50^ Heatmaps were visualized using the ComplexHeatmap package,^39^ with rows (genes) clustered with the Pearson distance metric and columns (samples) clustered with the Euclidean distance metric, using complete linkage clustering for both. The number of clusters to group the displayed genes was determined using the K‐means function in base R,^50^ with visualization of the total sum of squares as an elbow plot and average silhouettes in a silhouette plot. The number of clusters to group the samples (columns) was selected to enhance visualization. For barplots of individual gene expression values, read counts were transformed using the GeTMM method^51^ and converted to counts per million using calcNormFactors and cpm commands in edgeR.^52^ Displayed log_2_ fold changes were derived from DESeq2 modeled data. In this method, the log fold changes are shrunken to prevent overestimating fold changes for genes with low counts and/or high dispersion. Enterocyte and enteroendocrine cell markers were referenced from previous work by Haber and colleagues.^53^

### 2.7 Gene annotations

Annotations for select hormone‐related genes were taken from GeneCards^48^ (www.genecards.org) and the literature: AGT,^54^, ^55^ ARHGEF25,^56^ CCK,^57^, ^58^, ^59^ GAST,^57^, ^60^, ^61^ GHRL and GHRLOS,^62^, ^63^, ^64^ GIP,^60^ MLN,^57^, ^60^ NPW,^65^, ^66^, ^67^ NPY,^68^, ^69^ SST,^70^, ^71^, ^72^ DRD1,^73^ NRG4,^74^, ^75^, ^76^ NTSR1,^60^, ^77^ TAC3,^68^, ^78^, ^79^ AVP,^80^, ^81^, ^82^, ^83^, ^84^, ^85^ C1QTNF12,^86^, ^87^ LHB,^88^, ^89^ NTS,^60^, ^77^ OXT,^8^, ^84^, ^85^, ^90^, ^91^ SCTR,^92^ PAQR5,^93^ P2RY1,^94^ RARB.^95^ Annotations for select immune and stress response genes were taken from GeneCards^48^ (www.genecards.org).

### 2.8 Human tissue

Human intestinal tissue was acquired from the organ donation group LifeGift within the Texas Medical Center (USA). All organ donors were adults not presenting with any known gastrointestinal disease, surgery, or trauma. Individuals positive for hepatitis B or C, HIV, or COVID were excluded. Tissue was delivered to the lab within ~4 h of the patient initiating organ harvest and within ~1 h of the harvest of the gastrointestinal tract.

### 2.9 Hormone secretion

To measure secreted hormones from the treated organoids, supernatants from the apical (or basolateral, where noted) side of the transwells were assessed using the Luminex MILLIPLEX Human Metabolic Hormone kit (EMD Millipore, USA) or using a serotonin ELISA (SER39‐K01, Eagle Biosciences, USA). For measuring hormones secreted from whole human tissue, approximately 2 cm by 2 to 3 cm pieces of human tissue were incubated in 5 mL of *L. reuteri* conditioned media or media control in 6 well plates for 3 h at 5% CO_2_. AVP was measured with the Arg8‐Vasopressin ELISA kit (ADI‐900‐017A, Enzo, USA), LHB with the Luteinizing Hormone (hLH) ELISA Assay kit (HLH31‐K01, Eagle Biosciences, USA), adipolin with the Human CTRP12 ELISA kit (SK00392‐06, Aviscera Bioscience, USA), and kisspeptin with the Human Kisspeptin ELISA kit (ab288589, Abcam, USA). For organoids, statistical significance was determined using a one‐way ANOVA followed by a Dunnett's test with the LDM4 treatment used as the control. For human tissue, data were modeled with linear mixed models with the human patient included as a random variable using the lmer function of the lme4^96^ package with REML = FALSE and the control optimizer = “bobyqa”. Subsequently, statistical significance was determined using the emmeans function^97^ with a Benjamini–Hochberg multiple testing correction.

### 2.10 Single‐cell RNA‐Sequencing analysis

Single‐cell RNA‐Sequencing (scRNA‐Seq) analysis of the Human Gut Atlas (https://www.gutcellatlas.org/, adult epithelium, jejunum) was performed as previously described.^33^ Briefly, scRNA‐Seq data from the adult jejunum were analyzed using the Seurat package in R (v 5.0.3). After data normalization, data clustering, and UMAP generation, genes of interest were plotted using the FeaturePlot function.

### 2.11 Immunofluorescence

Adipolin was visualized on human jejunal tissue from organ donors as previously described^33^ using the antibody (NBP1‐90700, Novus Biologicals, Centennial, CO, USA) diluted to between 1:20 and 1:50 and detected with Rhodamine Red™‐X (AB_2338028, Jackson ImmunoResearch, West Grove, PA, USA) diluted to 1:200. An E‐cadherin conjugated antibody (1:50, 560062, BD Pharmingen, Franklin Lakes, NJ, USA) and DAPI (1:10, NucBlue™ Fixed Cell Stain ReadyProbes™ reagent, R37606, Invitrogen, USA) were applied simultaneously with Rhodamine Red™‐X.

### 2.12 Metabolomics

Separate from that utilized for HIO experiments, two batch preparations of LDM4 media and conditioned media generated from *L. reuteri* 6475 and 17938 grown in these LDM4 batches were sent to Metabolon (Morrisville, NC, USA) to be analyzed using their Global Metabolomics pipeline. From the conditioned media and LDM4 media, 257 identified compounds were detected across the samples. Metabolite peak areas were calculated from total ion counts integrated from the area under the curve. Data were prepared as normalized (median‐scaled per metabolite), normalized imputed data (normalized data where missing values were replaced with the lowest value per metabolite), and natural log‐transformed normalized imputed data. Natural log‐transformed normalized imputed data were used to calculate fold changes between sample groups and were used to calculate significance via an ANOVA (aov function in R) between groups. The resulting *p*‐values were adjusted across all comparisons and metabolites using the Benjamini–Hochberg procedure to produce *q*‐values.

Normalized and imputed data were visualized in a principal coordinate analysis (multi‐dimensional scaling, MDS) using a Bray‐Curtis distance matrix in R using the phyloseq package (v1.42.0).^98^ The contribution of sample variables to the variation in the data was modeled using a PERMANOVA of the form: Bray‐Curtis distance matrix ~ treatment + media batch using the adonis2 function in vegan (v2.6–6.1).^38^ Correlation analyses were performed on these data using the cor function with a Spearman correlation from the stats package.^50^ Differences between means (circle size) and adjusted *p*‐values (circle color) from a Wilcoxon test with a Holm correction were visualized as a correlogram using the ComplexHeatmap package (v2.14.0).^39^ The significance of differences in metabolite fold changes between sample groups was calculated using a Wilcoxon test. Significantly different metabolites were called at *p* < .05 and *q* < .05. Normalized but not imputed data were visualized as a heatmap using the ComplexHeatmap package,^39^ with rows (metabolites) grouped by metabolic functional annotations provided by Metabolon and columns (samples) clustered with the Euclidean distance metric using complete linkage clustering.

## 3 RESULTS

### 3.1 *NGN3* ‐HIOs facilitate the study of *L. reuteri*'s interactions with the enteroendocrine system

**FIGURE 1:**
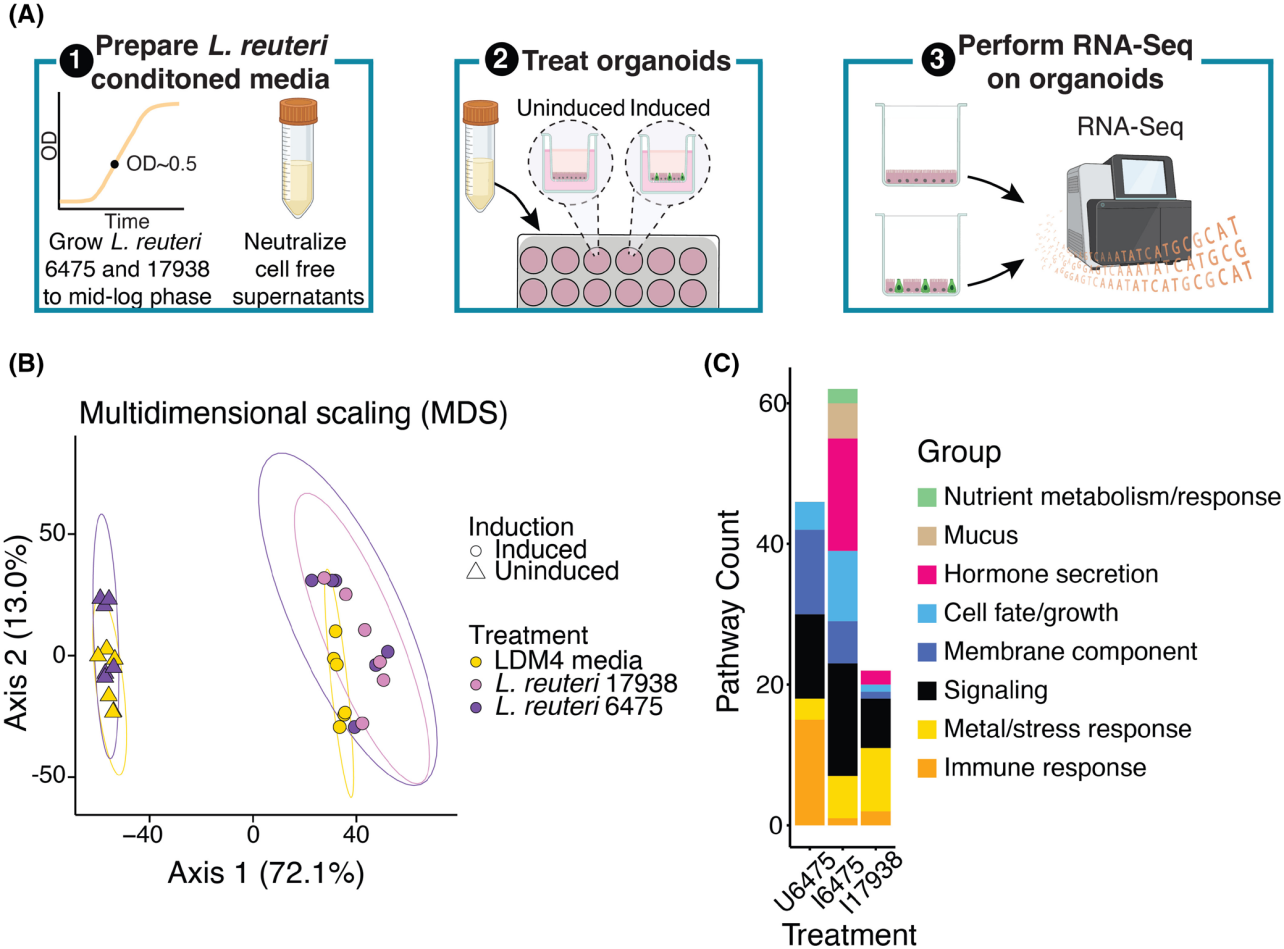
Induced and uninduced *NGN3*‐HIOs differentially respond to *L. reuteri* treatment. (A) Overview of RNA‐Seq experiment. (1) *L. reuteri* conditioned media were prepared by growing *L. reuteri* 6475 and 17938 in LDM4 media to mid‐log phase. The bacterial cells were spun out. The resulting conditioned media were brought to neutral pH and rendered cell‐free by filtration. The conditioned media were then lyophilized and resuspended in HIO differentiation media. (2) These treatments were then placed onto uninduced or induced *NGN3*‐HIOs in transwells for 3 h. (3) The organoid cells were harvested, and the isolated RNA was sent for RNA‐Seq. Created with BioRender.com. (B) Multidimensional scaling of transcriptomic data using a Euclidean distance metric from *NGN3*‐HIOs induced or uninduced and treated with *L. reuteri* 6475, 17938, or LDM4 media control. Ellipses for illustration purposes are modeled from the data following a *t*‐distribution. (C) Enriched functional categories of differentially expressed genes in *L. reuteri* treatments over media alone. U6475 is *L. reuteri* 6475 versus media control in uninduced *NGN3*‐HIOs. I6475 is *L. reuteri* 6475 versus media control in induced *NGN3*‐HIOs. I17938 is *L. reuteri* 17938 versus media control in induced *NGN3*‐HIOs. Some functional groups are listed as belonging to two categories (see Table S3 for further details).

To determine how *L. reuteri* strains 6475 and 17938 affect the intestinal epithelium, we designed an RNA‐Seq experiment using human intestinal organoids (HIOs) treated with pH‐neutralized conditioned media produced by these strains in log phase, when the bacteria are expected to be highly metabolically active (Figure 1A). The media thereby represent any products the *L. reuteri* strains released into their growth media. The specific HIOs we utilized originated from adult jejunal stem cells and have been engineered for the inducible expression of the transcription factor *NGN3*.^32^ *NGN3* induction results in HIOs enriched in enteroendocrine cells with a decrease in the relative abundance of enterocytes.^32^ We have previously characterized this HIO line and observed that when induced, this line displays little change in epithelial cell types with the exception of enterocytes, which decrease in abundance, and enteroendocrine cells, which increase in abundance.^32^ As well, all of the known subtypes of enteroendocrine cells were observed to be present in these organoids.^32^ Hence, with this *NGN3*‐HIO line we can measure the effects of the *L. reuteri* strains on induced *NGN3*‐HIOs enriched in enteroendocrine cells and on uninduced *NGN3*‐HIOs primarily comprised of enterocytes. We performed our treatments for 3 h, as we have previously observed that this HIO line is capable of secreting hormones glucose‐dependent insulinotropic peptide (GIP)^32^ and oxytocin^33^ in response to stimuli within this time.

We tested *L. reuteri* 6475 on uninduced *NGN3*‐HIOs (~90% enterocytes, <2% enteroendocrine cells)^32^ and *L. reuteri* 6475 and 17938 on enteroendocrine‐enriched (induced) *NGN3*‐HIOs (~50% enterocytes, ~40% enteroendocrine cells).^32^ *L. reuteri* 17938 was not tested on uninduced HIOs. RNA‐Seq of the organoids produced an average of 16.1 million reads per library (Tables 1 and S1). To confirm that induced *NGN3*‐HIOs were enriched in enteroendocrine cells and depleted in enterocytes compared to uninduced *NGN3*‐HIOs, we checked the expression levels of known enterocyte and enteroendocrine cell markers^53^ (Figures S1 and S2). Gene expression levels followed the expected patterns, with enterocyte markers being downregulated and enteroendocrine markers upregulated with *NGN3* induction (Figures S1 and S2).

**TABLE 1:** Summary of RNA‐Seq libraries.

HIO type	Treatment	Number of HIO experiments	Number of replicate HIOs within an experiment	Total number of RNA‐Seq libraries	Average read count
Uninduced	LDM4	2	3	6	18 968 834.33
Uninduced	*L. reuteri* 6475	2	3	6	15 465 905.00
Enteroendocrine‐enriched	LDM4	2	3	6	15 972 039.33
Enteroendocrine‐enriched	*L. reuteri* 6475	2	3	6	14 287 207.00
Enteroendocrine‐enriched	*L. reuteri* 17938	2	3	6	15 720 129.33

*Note*: Read counts shown are post‐filtering and alignment to the human genome (see Methods). See Table S1 for further details.

To globally assess whether the HIOs were impacted by the *L. reuteri* conditioned media, we performed an unsupervised analysis using dimensionality reduction with multidimensional scaling (MDS) produced from a Euclidean distance matrix of the gene expression data. As expected, the MDS plot illustrated that the data could be separated in dimension one by whether the HIOs were induced for *NGN3* expression or not, indicating *NGN3* induction was likely the greatest contributor to the variation in global gene expression (Figure 1B). To quantify the contribution of induction and the contributions of *L. reuteri* treatments and biological replication, we performed a PERMANOVA on the Euclidean distance matrix. Our PERMANOVA model reported that *NGN3* induction explains 70.8% of the variation (pseudo‐*F* = 120.763, *p* = .001), biological replication 9.2% of the variation (pseudo‐*F* = 15.685, *p* = .001), *L. reuteri* treatment 4.4% of the variation (pseudo‐*F* = 3.768; *p* = .011), and that the interaction of treatment and induction was not significant (1.5% of the variation; pseudo‐*F* = 2.578; *p* = .082). Similar results were obtained using the Jaccard similarity index. These results indicated that most of the variation in data resulted from *NGN3* induction and that the addition of *L. reuteri* 6475 or 17938 had a relatively smaller but still significant effect on HIO gene expression.

To gain further insight into the variation in gene expression in our data, we investigated gene expression correlations among pairwise comparisons of samples. We observed that induced HIOs treated with either *L. reuteri* strain were significantly less correlated than *L. reuteri* 6475 versus media control on uninduced HIOs (Figure S3A,B). We also observed that the correlations between induced HIOs treated with *L. reuteri* 6475 versus their media controls compared to those treated with *L. reuteri* 17938 versus their media controls were similar (*p* = .09), although the mean correlation for induced HIOs treated with *L. reuteri* 6475 versus their media controls was lower (Figure S3A,B). Together, these results further support that both *L. reuteri* strains significantly altered HIO gene expression when the HIOs were induced and suggest that the *L. reuteri* strains similarly affected gene expression.

### 3.2 *L. reuteri* strains 6475 and 17938 impact the expression of hormone, nutrient, mucus, metal/stress response, and immune‐related genes in native and/or enteroendocrine‐enriched HIOs

We next sought to determine the genes impacted by *L. reuteri* strains 6475 and 17938 in the induced and uninduced *NGN3* HIOs. We identified differentially expressed genes (DEGs) between these two strains and across the induction state of the HIOs. Specifically, we compared the effect of *L. reuteri* 6475 in the uninduced and induced states to their media controls and *L. reuteri* 17938 in the induced state to its media control. We found a similar number of genes impacted by *L. reuteri* 6475 in induced and uninduced HIOs, but fewer DEGs by *L. reuteri* 17938 in induced HIOs (Tables 2 and S2). While at first glance, this may suggest *L. reuteri* 6475 affects HIOs differently than 17938, only 12 genes were differentially expressed between *L. reuteri* 6475 and 17938 in induced HIOs (Table 2 and Figure S3C). On investigating the gene expression data, we observed that *L. reuteri* 17938 largely affects gene expression in the same direction as 6475; however, the fold change in gene expression for 17938 failed to pass our significance thresholds. These results reinforce those of our correlation analysis (Figure S3), suggesting that though *L. reuteri* 6475 had a more potent effect on transcriptional change in our induced HIOs in this experimental setup, the two strains had largely similar effects on gene transcription.

**TABLE 2:** Summary of genes differentially regulated between *L. reuteri* strains 6475 and 17938 in induced and uninduced HIOs.

Comparison	Upregulated	Downregulated
U6475‐ULDM4	359	148
I6475‐ILDM4	307	189
I17938‐ILDM4	66	62
I6475‐I17938	1	11

*Note*: Groups are labeled as “U” for uninduced, “I” for induced, “6475” for treatment with *L. reuteri* 6475 conditioned medium, “17938” for treatment with *L. reuteri* 17938 conditioned medium, and “LDM4” for treatment with bacterial growth medium.

To determine how these transcriptional changes might functionally affect the HIOs, we looked for functional enrichments in the DEGs. Using the PANTHER classification system^47^ and the Reactome annotated pathways,^45^, ^46^ we identified enriched functional annotations within the sets of DEGs. Broadly across all datasets, the *L. reuteri* DEGs were enriched in functions regarding response to the environment. These functions included nutrient, stress, metal, and immune response, and cell fate/growth, membrane components, and signal transduction (Figure 1C and Table S3). As anticipated, the induced HIOs treated with either *L. reuteri* strain were also enriched in genes for hormone secretion (Figure 1C and Table S3). The induced cells treated with *L. reuteri* 6475 were additionally enriched for genes relating to mucus.

To further investigate and understand the DEGs and their regulation, we annotated these genes within the functional groups and looked for similar expression patterns and functions (Table S3). We were able to group the genes into eight clusters using K‐means clustering (Figure S4). These clusters represent genes with similar transcriptional responses to induction and the presence of *L. reuteri* and, therefore, may share similar regulatory mechanisms. For instance, genes within a cluster may share a transcription factor or be localized within the same cell type. As cell types within the small intestine have partially non‐overlapping functions,^99^ this scenario would promote clusters being enriched in one or two closely related functions.

Indeed, we observed this to be the case (Table 3): clusters were either enriched in one or two related functions or were not enriched in any function. Clusters 1 and 5 were enriched in genes involved in hormone secretion, cluster 2 in cell adhesion, cluster 3 in stress/immune response, cluster 6 in nutrient response, and clusters 7 and 8 in mucus genes. Therefore, the clusters generated by our heatmap are consistent with gene clusters of related functionalities, perhaps from genes expressed in the same or similar cell types.

**TABLE 3:** Functional enrichments within clusters of similarly expressed DEGs.

Cluster	U6475‐ULMD4	I6475‐ILDM4	I17938‐ILDM4	I6475‐U6475	ILDM4‐ULDM4	Cell adhesion	Cell fate	Hormone secretion	Mucus	Nutrient response	Signaling	Stress/Immune response
1	−	+		+	+	0.454	0.793	0.094*	NA	0.542	< 0.001***	1
2	−	−	−	+	+	0.064*	0.879	0.879	0.231	0.542	0.542	0.454
3	+	+	+	+	+	NA	0.82	0.454	NA	0.82	0.879	0.021*
4	+			−		NA	0.106	NA	NA	0.542	0.399	0.655
5	+	+	+	−	−	NA	0.542	0.064*	NA	0.283	NA	0.454
6	−			−	−	NA	0.454	0.769	NA	0.037*	NA	0.399
7		−	−	−	−	0.231	0.454	0.879	0.064*	0.879	0.542	0.542
8		−				NA	0.283	0.769	0.086*	NA	0.772	0.399

*Note*: Clusters are listed as in Figure S4. Columns U6475‐ULDM4 through ILDM4‐ULDM4 summarize whether genes within that cluster are predominantly up (+) or down (−) regulated for the given comparison. FDR corrected significance values for functional groups: *q < .1; **q < .01; ***q < .001. Values with *q* < .1 are bolded.

### 3.3 *L. reuteri* impacts on immune and stress response

To see if our data are consistent with the known functions of *L. reuteri* on the intestinal epithelium, we first investigated the immune and stress response DEGs. We observed that many immune‐related genes were downregulated, and a few metal and stress response genes were upregulated by *L. reuteri* 6475 (Figure S5A). Tumor necrosis factor (TNF), which *L. reuteri* 6475 has been previously observed to downregulate^17^ and suppress,^3^ was not expressed in our HIOs; however, TNFSF15, which is induced by TNF and activates NF‐kappaB,^100^ was decreased in induced HIOs treated with *L. reuteri* 6475. Consistent with *L. reuteri* 6475 mediated suppression of NF‐kappaB and inflammatory responses,^17^ several chemokines were downregulated by *L. reuteri* 6475: IL‐8 (CXCL8), CCL2 (MCP‐1), CXCL2, CX3CL1, and CXCL3. Secreted MCP‐1, we observe, was also repressed by both *L. reuteri* strains only on induced *NGN3*‐HIOs (Figure S5B), suggesting a role of enteroendocrine cells in downregulating inflammatory responses. TLR4, which senses stimuli and upregulates inflammatory responses,^101^ was also downregulated by *L. reuteri* 6475. *L. reuteri* 6475 additionally upregulated interleukin 18 binding protein (IL18BP), an inhibitor of the proinflammatory IL‐18.^102^ Defensin‐6, interferon epsilon, several metallothionein genes, and aquaporin‐1 and ‐7, which respond to environmental changes, were upregulated by *L. reuteri* 6475. These data are consistent with reports of *L. reuteri* 6475 having anti‐inflammatory, immune modulatory, and stress response effects on the gut epithelium. *L. reuteri* 17938 had a less pronounced effect on immune and stress response genes. None of the chemokine or aquaporin genes were significantly impacted, and only about half of the metallothioneins were differentially regulated in response to *L. reuteri* 17938. As previously mentioned, these results are largely due to *L. reuteri* 17938 impacting gene expression in the same direction but not the same magnitude as 6475 in our experiment.

### 3.4 *L. reuteri* affects the transcription and secretion of enteroendocrine cell hormones

**FIGURE 2:**
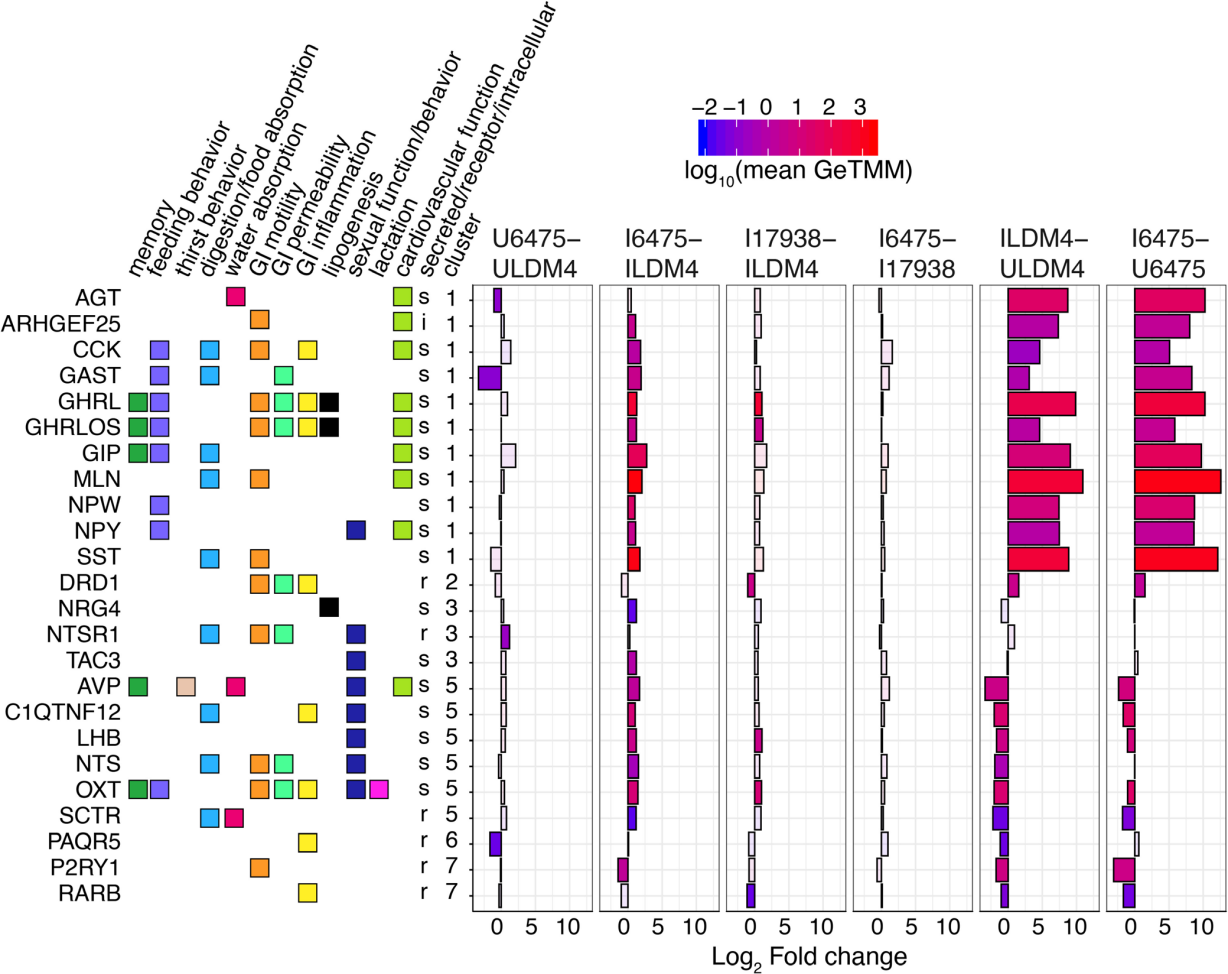
Hormone genes differentially expressed by *L. reuteri*. DEGs annotated as having hormonal function are shown. The genes are annotated with their function, whether they are secreted (s), a receptor (r), or intercellular (i), and the cluster to which they belong, as in Figure S4. The plot shows the log_2_ fold change expression of the gene for the indicated comparison. The bars are colored using the log_10_ scaled mean GeTMM counts to illustrate how abundantly expressed the gene is. Transparent overlays are used for genes not differentially expressed for the given comparison. Comparisons shown: U6475‐ULDM4, *L. reuteri* 6475 on uninduced HIOs compared to LDM4 media control; I6475‐ILDM4, *L. reuteri* 6475 on induced HIOs compared to LDM4 media control; I17938‐ILDM4, *L. reuteri* 17938 on induced HIOs compared to LDM4 media control; I6475‐I17938 *L. reuteri* 6475 compared to *L. reuteri* 17938 on induced HIOs; ILDM4‐ULDM4, LDM4 media control on induced versus uninduced HIOs; I6475‐U6475, *L. reuteri* 6475 on induced versus uninduced HIOs. For each, positive fold changes indicate genes upregulated by the condition listed first.

We next focused on clusters 1 and 5 for their enrichment of hormone genes (Figure 2). Cluster 1 appears as we would expect for canonical gut hormones derived from enteroendocrine cells: the genes in cluster 1 increased in expression with *NGN3* induction. These genes included those for the hormones angiotensinogen (AGT), cholecystokinin (CCK), gastrin (GAST), ghrelin (GHRL and GHRLOS), gastric inhibitory polypeptide aka glucose‐dependent insulinotropic polypeptide (GIP), motilin (MLN), neuropeptide W (NPW), neuropeptide Y (NPY), and somatostatin (SST). Except for *AGT*, all genes were significantly upregulated by *L. reuteri* 6475. Only *GHRL* and *GHRLOS* were significantly upregulated by *L. reuteri* 17938.

**FIGURE 3:**
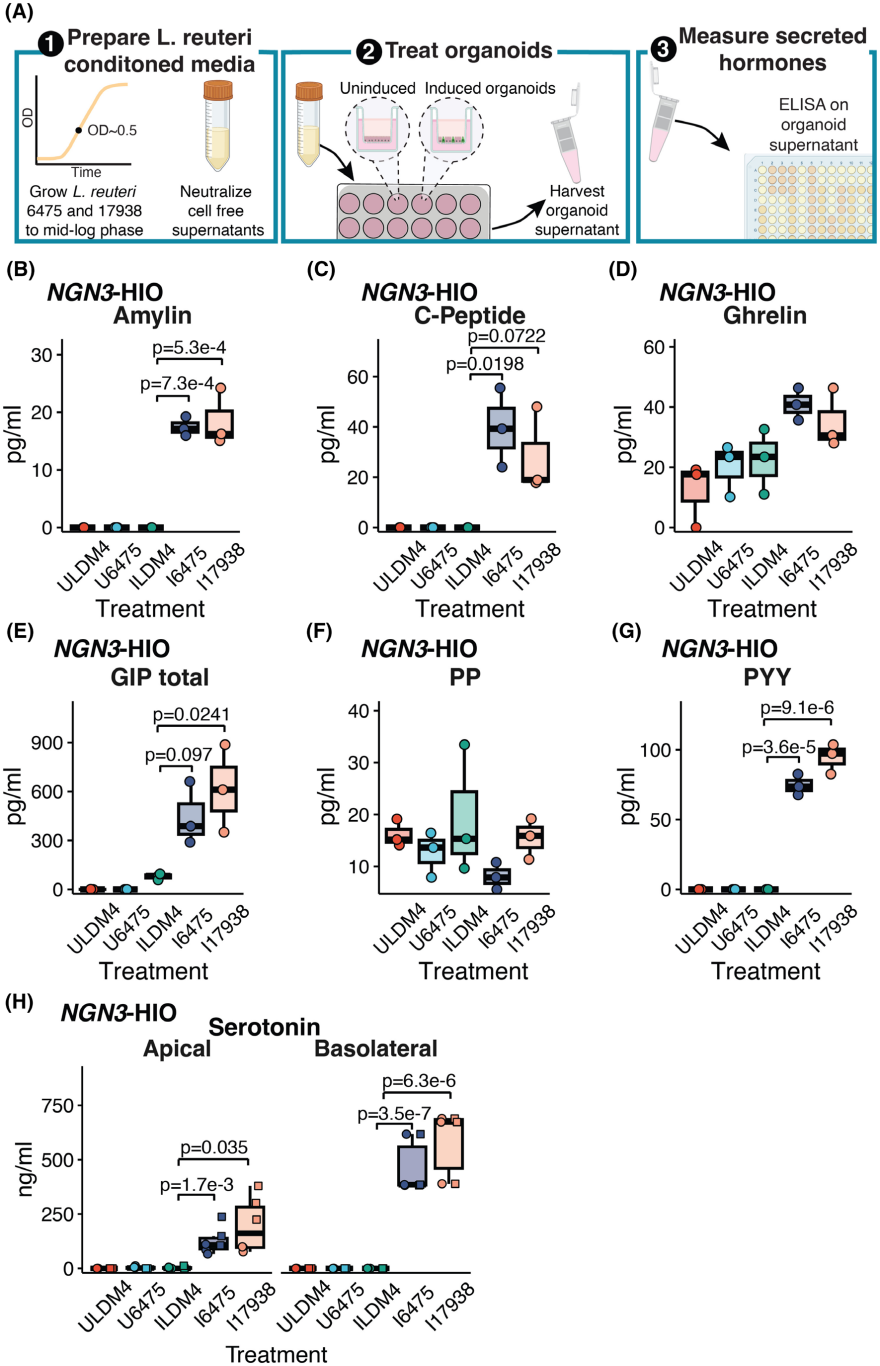
*L. reuteri* promotes the secretion of known enteroendocrine‐derived intestinal hormones. (A) (1) In order to measure the release of intestinal hormones from human intestinal organoids (HIOs), *L. reuteri* conditioned media are generated from mid‐log phase cultures of *L. reuteri*. These cultures are pH neutralized and rendered cell‐free. (2) *L. reuteri* conditioned media are then placed onto uninduced or induced *NGN3*‐HIOs. (3) Following incubation on the HIOs, the supernatant is collected and secreted hormones are measured by an ELISA. Created with BioRender.com. Secreted amylin (B), C‐peptide (C), ghrelin (D), GIP (E), PP (F), and PYY (G) measured from uninduced and induced *NGN3*‐HIOs in response to *L. reuteri* 6475 or 17938 conditioned media. Hormones in (B–G) were measured on the apical side only of the transwell. In (B–G), batches A and B from the RNA‐Seq experiment were pooled—each plot point represents the result of two organoid batches pooled together. (H) Serotonin released from the apical or basolateral side (as indicated) from uninduced and induced *NGN3*‐HIOs in response to *L. reuteri* 6475 or 17938 conditioned media. In H, shape denotes independent batches of organoids. Only *p*‐values <.1 are shown, with *p* < .05 being considered significant. Significance was determined using a Dunnett's test.

To determine if some of these gene expression differences might lead to differences in hormone secretion, we tested the organoid supernatant collected after applying *L. reuteri* 6475 and 17938 conditioned media to uninduced and induced *NGN3*‐HIOs. The harvested supernatants from the organoids were run on a Luminex panel consisting of metabolic‐related hormones (see Methods) (Figure 3A). From this panel, we were able to obtain measurable values of amylin, C‐peptide, ghrelin, GIP (total), pancreatic polypeptide (PP), and peptide YY (PYY) (Figure 3B–G). For amylin and PYY, both *L. reuteri* strains significantly increased the secretion of these hormones from induced *NGN3*‐HIOs (Figure 3B,G). The secretion of GIP was enhanced significantly (at *p* < .05) by *L. reuteri* 17938, and C‐peptide secretion was significantly promoted by *L. reuteri* 6475; although for both hormones, the other *L. reuteri* strain promoted secretion at *p* < .1 (Figure 3C,E). PYY, whose secretion was promoted, was not transcriptionally upregulated by either *L. reuteri* strain. PP (*PPY*), amylin (*IAPP*), and insulin (*INS*) gene counts were below the detection limit in the RNA‐Seq data.

Interestingly, no genes related to serotonin metabolism or transporters (*TPH1, TPH2, DDC, SLC18A1, SERT*) were altered by either *L. reuteri* strain. Nevertheless, we observed that *L. reuteri* 6475 and 17938 promote serotonin secretion (Figure 3H). Collectively, these data indicate that *L. reuteri* regulates numerous gut hormones; however, *L. reuteri* may upregulate either or both the expression and secretion of intestinal hormones.

### 3.5 *L. reuteri* affects the transcription and secretion of enterocytic hormones

While the genes in cluster 1 were upregulated by *NGN3* induction, those in cluster 5 were downregulated by *NGN3* induction (Figure 2). The genes downregulated were for hormones vasopressin (AVP), adipolin (C1QTNF12), luteinizing hormone subunit B (LHB), neurotensin (NTS), and oxytocin (OXT). Neuregulin‐4 (*NRG4*) and tachykinin‐3 (*TAC3*) were unaffected by induction. All these hormone genes were significantly upregulated by *L. reuteri* 6475, while only *LHB* and *OXT* were significantly upregulated by *L. reuteri* 17938. Interestingly, among these hormones, only neurotensin is well established to be produced by the gut epithelium. In mice, neurotensin is observed within villus proximal enteroendocrine L‐cells^103^, ^104^ and is thought to be produced in L cells only after they have migrated away from crypts and are exposed to increasing levels of BMP4 signaling.^103^

**FIGURE 4:**
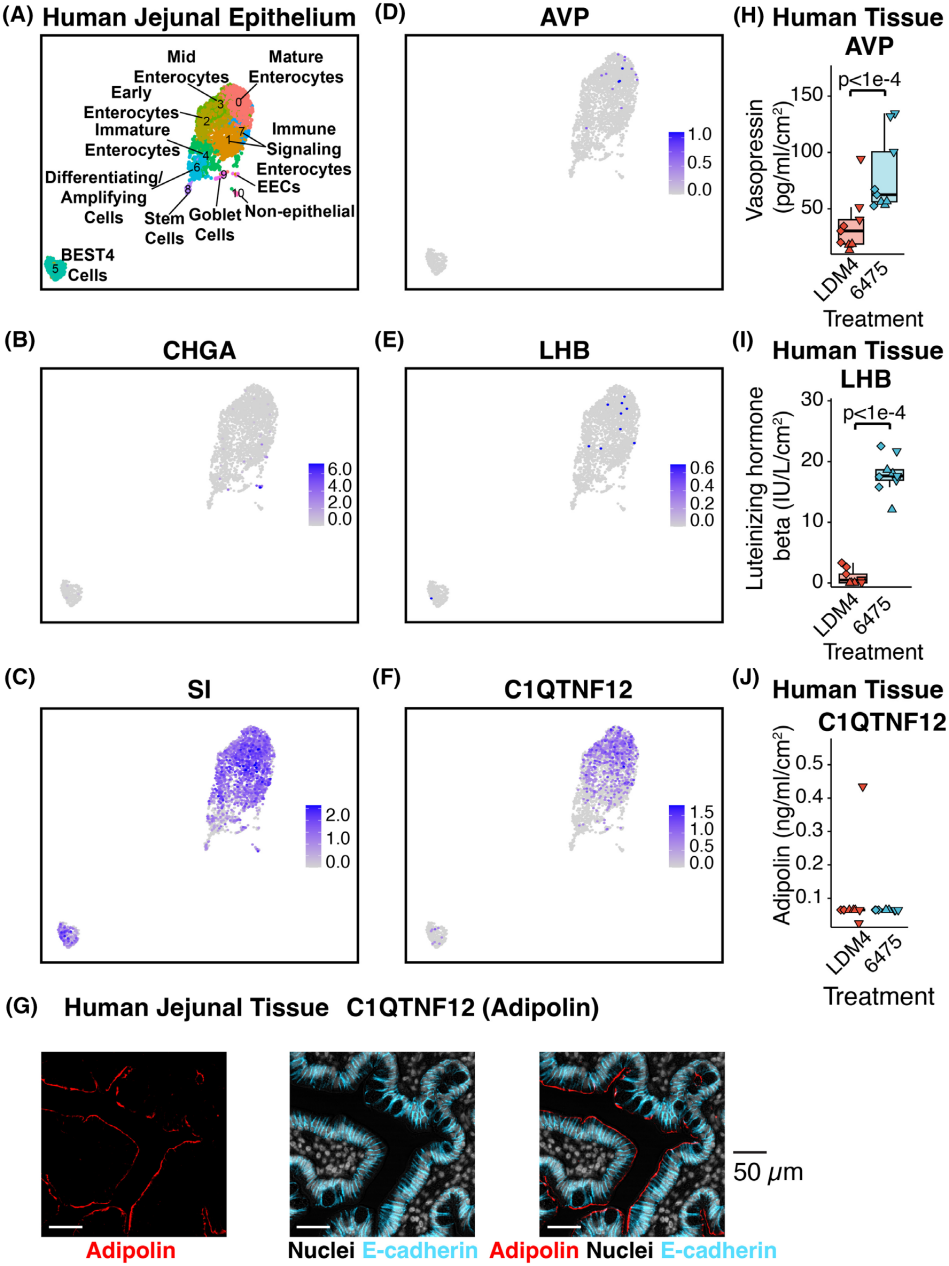
*L. reuteri* promotes the secretion of novel enterocytic hormones. (A) Gut Cell Atlas annotated UMAP of the adult jejunum (adapted from Danhof et al.^33^), highlighting the enteroendocrine marker *CHGA* (B), the enterocyte marker *SI* (C), vasopressin (*AVP*, D), luteinizing hormone subunit beta (*LHB*, E), and adipolin (*C1QTNF12*, F). (G) Adipolin visualized in human jejunal tissue. The scale bar represents 50 μm. Secretion of (H) vasopressin, (I) luteinizing hormone subunit beta, and (J) the lack of secretion of adipolin from whole human jejunal tissue using the method shown in Figure 3A except with ex vivo human jejunal intestinal tissue. Shape represents unique human intestinal donors. Significance was determined using a linear mixed model with *p* < .05 considered significant. Only *p*‐values <.05 are shown.

Recently, we reported that oxytocin is produced by enterocytes in the small intestinal epithelium, and *L. reuteri* promotes its secretion.^33^ To determine if enterocytes also produce any of these hormones, we analyzed the adult jejunum single‐cell RNA‐Seq (scRNA‐Seq) data within the Gut Cell Atlas.^105^ While chromogranin A (*CHGA*) transcription clustered with enteroendocrine cells, transcription of *AVP*, *LHB*, and *C1QTNF12* (adipolin) clustered similarly to that for sucrose isomaltase (*SI*), a marker of enterocytes (Figure 4A–F). Furthermore, we confirmed by immunofluorescence that *C1QTNF12* (adipolin) is produced in enterocytes in the human jejunum (Figure 4G).

Next, we checked if *L. reuteri* induces the secretion of any of these hormones from whole intestinal tissue as it does for oxytocin.^33^ *L. reuteri* induced the release of vasopressin and LHB but not adipolin from the human jejunum (Figure 4H–J). Given that *AVP* and *LHB* transcripts are enriched in epithelial cells in adult gut tissue^105^ (*p* = 4.1e−3 for *AVP* in the epithelium across the entire adult intestine, *p* = 0 for just jejunum; *p* = 1.0e−5 for *LHB* in the epithelium across the entire adult intestine, *p* = .014 for just jejunum, hypergeometric distribution), the released vasopressin and LHB may originate from the epithelium rather than other regions of the intestinal tissue.

In looking at the functions of the hormones in cluster 5, these hormones have roles in sexual function and behavior. In contrast, those in cluster 1 have functions mostly in feeding behavior and cardiovascular function. We also noticed that kisspeptin (KISS1), a hormone characterized in the brain with roles in gonad development,^106^ though not differentially regulated by *L. reuteri*, was expressed in the *NGN3*‐HIOs and downregulated by induction. Like the other hormones in cluster 5, *KISS1* appears to be produced in enterocytes (Figure S6A). We looked to see if *L. reuteri* could induce its secretion and found no evidence of *L. reuteri* mediating the release of KISS1 (Figure S6B).

### 3.6 Metabolites produced by *L. reuteri* 6475 and 17938

**FIGURE 5:**
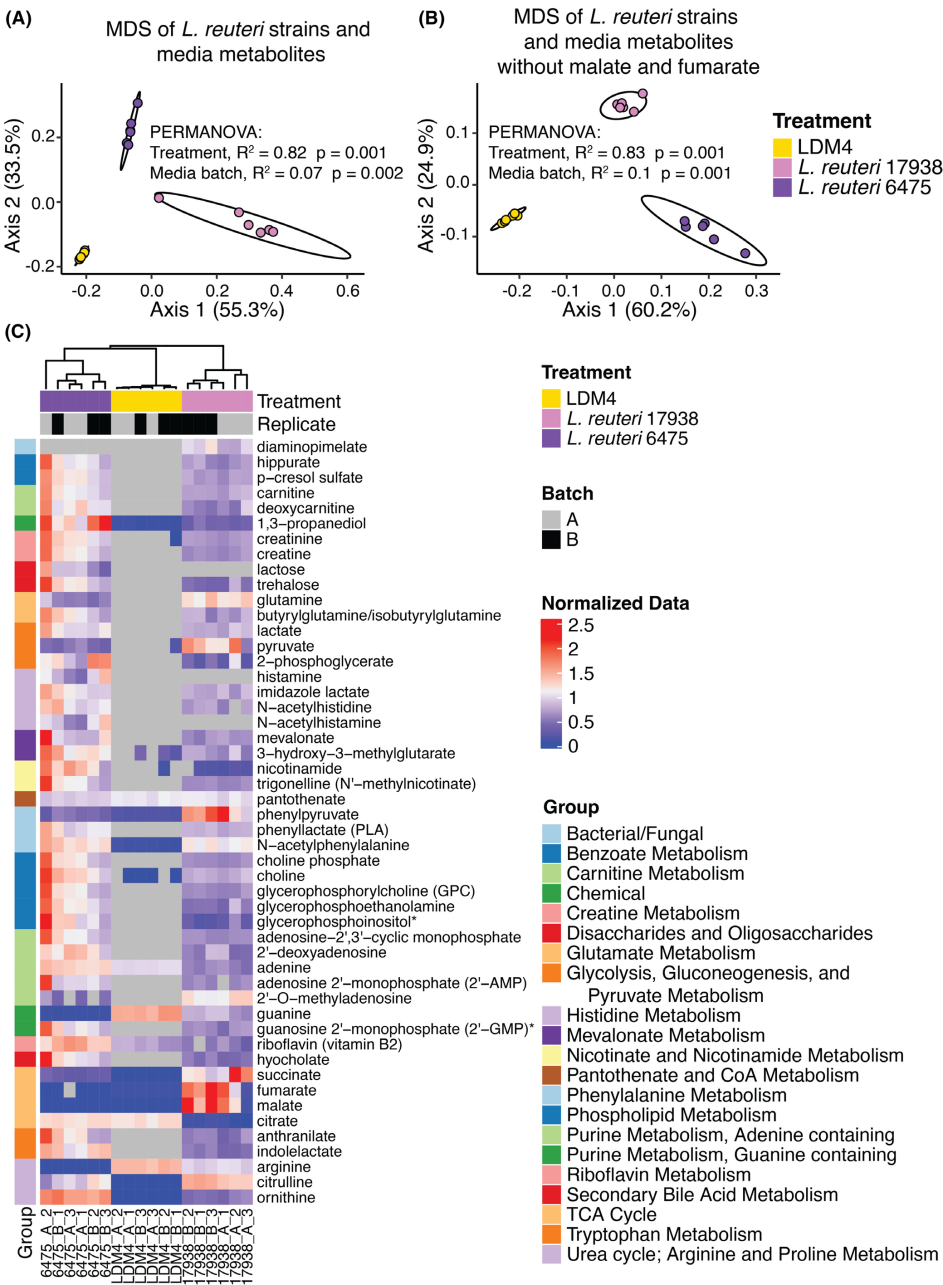
*L. reuteri* 6475 and 17938 conditioned media contain differential levels of metabolites. (A) Multidimensional scaling (MDS) using a Bray‐Curtis distance metric of the metabolome of *L. reuteri* conditioned media and the LDM4 media using normalized imputed data. Ellipses for illustration purposes are modeled from the data following a *t*‐distribution. (B) MDS as in A but with the metabolites fumarate and malate removed. (C) Heatmap of select metabolites significantly different between *L. reuteri* strains and from the media using normalized non‐imputed data. The color of the bar on the far left indicates the biochemical pathway to which the metabolites (rows) are associated. Samples (columns) are annotated by the batch of media from which they were prepared. Normalized heatmap values are shown on a red (high) to blue (low) scale, with gray used if the metabolite was not detected.  * Metabolite not confirmed based on a standard.

As a resource to begin understanding how these strains mediate these transcriptional and secretory effects on the epithelial layer, we generated metabolome datasets for both strains as well as for the LDM4 media (Tables S5–S9). As expected, the strains produced distinct sets of metabolites from each other and compared to the LDM4 media (PERMANOVA: *R* ^2^ = 0.82, pseudo‐*F* = 51.89, *p* = .001), with a minimal contribution of the batch of media utilized (PERMANOVA: *R* ^2^ = 0.07, pseudo‐*F* = 8.45, *p* = .002) (Figure 5A). While at first glance, *L. reuteri* 17938 appeared to separate more strongly from the LDM4 media along axis 1, this result was lost, and instead, *L. reuteri* 6475 was more separated from the LDM4 media if the metabolites fumarate and malate, which were produced at very high levels in *L. reuteri* 17938, were removed (Figure 5B). Correlation analysis with a Spearman correlation between and within these sample groups (using all metabolites) indicated that *L. reuteri* 6475 was less correlated with the LDM4 media than 17938 with LDM4 media (Figure S7A,B). Furthermore, *L. reuteri* 6475 had a greater number of differential metabolites over the media control (185) compared to 17938 (159) as well as had a significantly greater fold change of metabolites over media alone (Figure S7C).

Among the metabolites only significantly differential in *L. reuteri* 6475 compared to the media were histamine (produced) and other metabolites of histidine, lactose (produced) and lactate (produced), multiple nucleotides related to uracil and adenosine metabolism, and the B vitamin metabolite pantothenate (consumed) (Table S10). On the other hand, fewer metabolites were only significantly differential from the media in *L. reuteri* 17938. These include the tricarboxylic acid cycle (TCA) metabolite citrate (consumed), the peptidoglycan component diaminopimelate (produced), nucleotides xanthine (produced) and cytosine (consumed), ribitol (produced), and the glutamate metabolite (4‐hydroxyglutamate) (Table S11). As well, *L. reuteri* 17938 produced or consumed less of many metabolites including carnitine, creatine, nicotinamide, mevalonate, phenyllactate, choline, adenine, 2′‐deoxyadenosine, guanine, arginine, hypocholate, indolelactate, anthranilate, and ornithine (Figure 5C and Table S12). Such metabolites with lower production in *L. reuteri* 17938 could explain why *L. reuteri* 6475 had a greater effect on gene expression in the HIOs than *L. reuteri* 17938.

## 4 DISCUSSION

*L. reuteri* has been characterized as a beneficial microbe capable of affecting multiple aspects of host physiology within and beyond the gut. These effects are likely to involve host–microbe interactions that initiate at the intestinal epithelial layer. To begin to understand these interactions, here we used an organoid model enhanced in its number of enteroendocrine cells to study interactions between *L. reuteri* and intestinal hormones. While microbes have been identified that promote the release or expression of hormones or neuropeptides including GLP‐1,^107^, ^108^, ^109^ PYY,^108^, ^109^ serotonin,^107^, ^110^, ^111^, ^112^, ^113^ testosterone,^26^ and oxytocin,^33^ our study here focused on the effect of a single microbe on intestinal hormones using a human intestinal organoid model system. Our results indicate that multiple intestinal hormones are regulated by *L. reuteri* (Table 4); moreover, these data point towards there being novel hormones derived from enterocytes in the gut. Specifically, while luteinizing hormone subunit beta was previously observed as transcribed in the stomach and duodenum,^114^ and there is a report of vasopressin immunoreactivity in the human small intestine,^115^ these as well as kisspeptin and adipolin have not been described as intestinal epithelial hormones.

**TABLE 4:** Summary of *L. reuteri's* effects on gut hormones.

Hormone	Proposed or established hormone cell type	Expression	Secretion
Amylin	Enteroendocrine	ND	+ (this work)
C‐peptide	Enteroendocrine	ND	+ (this work)
CCK	Enteroendocrine	+ (this work)	ND
Gastrin	Enteroendocrine	+ (this work)	ND
Ghrelin	Enteroendocrine	+ (this work)	NS (this work)
GIP	Enteroendocrine	+ (this work)	+ (this work)
Luteinizing hormone, beta subunit	Enterocyte	+ (this work)	+ (this work)*
Motilin	Enteroendocrine	+ (this work)	ND
Neurotensin	Enteroendocrine	+ (this work)	ND
NPW	Enteroendocrine	+ (this work)	ND
NPY	Enteroendocrine	+ (this work)	ND
Oxytocin	Enterocyte	+ (this work)	+^33^
PYY	Enteroendocrine	NS (this work)	+ (this work)
Secretin	Enteroendocrine	NS (this work)	+^33^
Serotonin	Enteroendocrine	NS (this work)	+ (this work)
Somatostatin	Enteroendocrine	+ (this work)	ND
Vasopressin	Enterocyte	+ (this work)	+ (this work)*

Abbreviations: ND, not determined; NS, not significant; +, upregulated.

* Not confirmed if secretion occurs from epithelial cells.

While we found several well‐known intestinal hormones are not regulated by *L. reuteri* (including GLP‐1 and pancreatic peptide (PP)), we observed that *L. reuteri* largely transcriptionally upregulates gut hormones. We also found that *L. reuteri* promotes the secretion of several gut hormones. This study mainly focused on the effect of *L. reuteri* on hormones of the small intestine, where we postulate that *L. reuteri* may act therapeutically in humans. Hence, these data broadly suggest that *L. reuteri* acts beneficially by regulating intestinal hormones. Moreover, our study considered not just a single probiotic strain of *L. reuteri* but two different commercially used strains. Interestingly, our study failed to observe major differences between the two strains: *L. reuteri* 17938 appeared to transcriptionally affect HIOs enriched in enteroendocrine cells very similarly to *L. reuteri* 6475, albeit with a lower magnitude. Furthermore, the select hormones whose secretion we tested were similarly induced by both strains. In analyzing the metabolome of these strains, we observed that *L*. reuteri 17938 metabolized the bacterial growth media to a lesser extent than 6475, which could be responsible for *L. reuteri* 17938's lower effect on HIO transcripts.

Recently, several new enteric hormones have been described. In addition to the discovery of oxytocin in the intestinal epithelium, famsin,^116^ GDF15,^117^ and cholesin^118^ have been discovered. A survey of these peptide hormones in the Gut Cell Atlas^105^ suggests that, in addition to the previously described FGF19, guanylin, and uroguanylin,^31^ these hormones are made in enterocytes rather than enteroendocrine cells. The recognition that enterocytes can produce hormones has opened questions regarding the production of these hormones. Enteroendocrine cell‐derived hormones are produced from prohormones that are cleaved to the active hormone by prohormone convertases, some of which are exclusively produced in enteroendocrine cells.^119^ On stimulation, these hormones are subsequently secreted from vesicles stored in axon‐like structures within the cell.^120^ Hence, are these enterocytic hormones only processed by convertases made in enterocytes? Are the hormones stored in vesicles like those in enteroendocrine cells? And how and where are these vesicles released?

The function of these novel enterocytic hormones is additionally waiting to be determined. Non‐intestinal sources of oxytocin, vasopressin, kisspeptin, and luteinizing hormone have roles in regulating sexual function, and several also function in regulating eating or digestion. Famsin,^116^ GDF15,^117^ and cholesin^118^ have been characterized with roles related to metabolism and energy regulation. Given the known links between metabolic state and sexual function,^121^ potentially then, intestinal sources of oxytocin, vasopressin, kisspeptin, and luteinizing hormone might link metabolic state to sexual function.

Within the gut, these hormones most likely have local effects on the function of the gut in nutrient absorption, gut transit, epithelial cell homeostasis, and immune regulation. Total body loss of oxytocin^91^ and exogenous delivery of vasopressin have been demonstrated to alter water absorption and gut transit.^122^, ^123^, ^124^ Both hormones are also implicated as capable of regulating immune activity,^91^, ^125^, ^126^ and oxytocin is reported to promote intestinal proliferation and epithelial layer homeostasis.^91^, ^125^, ^127^ Kisspeptin is also reported to modulate gut motility, although this action has only been observed for centrally delivered kisspeptin.^128^ For luteinizing hormone, the situation is less clear as we were only able to detect the beta subunit of the hormone in the intestinal epithelium. A source of the alpha subunit (glycoprotein hormones, alpha polypeptide) in the intestine has yet to be reported.

How these hormones might signal from the epithelial layer to mediate any local or possible systemic effects is expected to be driven by their receptors. Like other hormones, they should be able to act in a paracrine mechanism on local receptors in the gut and act in an endocrine mechanism by entering circulation to target a receptor distal to the gut. Receptors for oxytocin (OXTR), vasopressin (AVPR1A, AVPR1B, AVPR2), luteinizing hormone (LHCGR), and kisspeptin (KISS1R) can be observed in human scRNA‐Seq data^105^ and other transcriptional datasets or by immunostaining as present on intestinal epithelial (OXTR, AVPR1B, KISS1R),^124^, ^126^, ^129^ mesenchymal (AVPR1A, LHCGR),^105^ and/or endothelial cells (OXTR, AVPR2)^105^ and on enteric neurons (OXTR, AVPR1B)^91^, ^105^, ^129^, ^130^ and immune cells (OXTR, KISS1R).^105^, ^131^ Given the innervation of the small intestine with the vagal nerve, signaling to receptors on enteric neurons could be a mechanism, in addition to circulation, by which these hormones affect systemic processes.

We also observed that adipolin is produced in the small intestinal epithelial layer. Adipolin has been observed as present in the small intestinal epithelium presented by the Human Protein Atlas (https://www.proteinatlas.org/ENSG00000184163‐C1QTNF12/tissue/small+intestine).^132^ In adipose tissue, adipolin was characterized as an adipokine that improves glucose tolerance and insulin response and reduces macrophages and proinflammatory immune responses.^86^ In the intestine, it may have similar immune and metabolic functions.

Previously, we determined that the hormone secretin is involved in *L. reuteri's* release of oxytocin.^33^ However, what *L. reuteri* makes to promote secretin's release is currently unknown. Presently, a variety of different microbial metabolites or structures have been shown to promote the release of or are associated with the release of intestinal hormones. These include short chain fatty acids,^133^, ^134^, ^135^ branched and aromatic amino acids,^135^ indoles,^136^ secondary bile acids,^137^ and microvesicles.^113^ Whether any of these molecules or others produced by *L. reuteri* are involved in the hormones affected here remains to be determined.

Our metabolomic analysis of the *L. reuteri* conditioned media provides numerous candidates for further investigation into how these strains interact with the intestinal epithelial layer. We expect that among the metabolites produced by both strains but in lower amounts in *L. reuteri* 17938 are those that regulate many of the gene expression effects we observe here. For example, phenyllactate^138^ and ornithine,^139^ which are produced by both strains but in higher amounts in *L. reuteri* 6475, have demonstrated physiological effects on the epithelial layer. Our metabolomic analyses also supported *L. reuteri* 6475 and not *L. reuteri* 17938 being able to produce histamine, *L. reuteri* 17938 having a partial TCA cycle,^28^ and both strains being able to liberate adenosine. We hope this resource will assist in the study of the biological effects of these strains.

Several limitations of our study design should be mentioned. First, the media conditions of the organoids have been observed to reduce inflammatory responses.^140^ Second, the organoids only represent the epithelial layer, so interactions between *L. reuteri* and the host that depend on immune cells, enteric neurons, or products of the lamina propria or circulation cannot be captured by this assay. While we anticipate some of these effects to be downstream of those mediated by the epithelial layer, interactions with the immune system that occur in parallel to effects on the epithelial layer are missing from these data. Third, the assay was performed using cell‐free supernatants with a three‐hour exposure. Hence, host responses that require intact structural components of *L. reuteri* or a different length of exposure are also not represented in this assay. In particular, interactions that occur from long‐term interactions between *L. reuteri* and the epithelial layer are missed in this assay. Fourth, the secretion assays were not designed to capture whether *L. reuteri* suppresses the secretion of hormones. Similarly, the transcriptomic data only consider *L. reuteri's* effect relative to bacterial growth media. Further follow‐up studies will be needed to determine if *L. reuteri* promotes the secretion of these hormones under more physiologically relevant conditions.

In conclusion, this work demonstrates that *L. reuteri* regulates several canonical and novel hormones of the intestinal epithelial layer. These results open exciting investigations regarding how *L. reuteri* influences a wide range of aspects of systemic physiology.

## AUTHOR CONTRIBUTIONS

Concept and design (Sara C. Di Rienzi, Heather A. Danhof, Micah D. Forshee, Robert A. Britton); intellectual contribution (Sara C. Di Rienzi, Heather A. Danhof, Micah D. Forshee, Robert A. Britton); data acquisition (Sara C. Di Rienzi, Heather A. Danhof, Micah D. Forshee); data analysis, statistical analysis, and interpretation (Sara C. Di Rienzi, Heather A. Danhof, Micah D. Forshee, Ari Roberts); drafting and editing manuscript (Sara C. Di Rienzi, Heather A. Danhof, Micah D. Forshee, Robert A. Britton); obtaining funding (Sara C. Di Rienzi, Heather A. Danhof, Robert A. Britton).

## FUNDING INFORMATION

U.S. National Library of Medicine [T15 LM007093] (SCD), National Institute of Allergy and Infectious Diseases [F32 AI136404] (HAD), Weston Family Foundation (SCD), BioGaia AB (RAB).

## DISCLOSURES

The authors declare no conflict of interest.

## Supporting information


Figures S1–S7.

Tables S1–S12.

## Data Availability

RNA‐Seq reads are available at NCBI GEO at https://www.ncbi.nlm.nih.gov/geo/ accession numbers GSE138350 and GSE268681. Metabolomic data are available in Tables S5–S12. Scripts for plots and data are available at https://github.com/sdirienzi/Lreuteri_HIORNASeq. An interactive ShinyApp displaying the RNA‐Seq data can be found here: https://sdirienzi.shinyapps.io/LreuHIORNASeq/.
