# Supplementary material for: Limosilactobacillus reuteri promotes the expression and secretion of enteroendocrine‐ and enterocyte‐derived hormones
Source: FASEB J. 2025 Mar 18;39(6):e70408. doi: 10.1096/fj.202401669R (PMC11914943; doi:10.1096/fj.202401669R)
Supplement: Supplementary file 1 — Figures S1–S7. [file FSB2-39-e70408-s002.pdf]

Supplemental Figure 1

Enterocyte markers

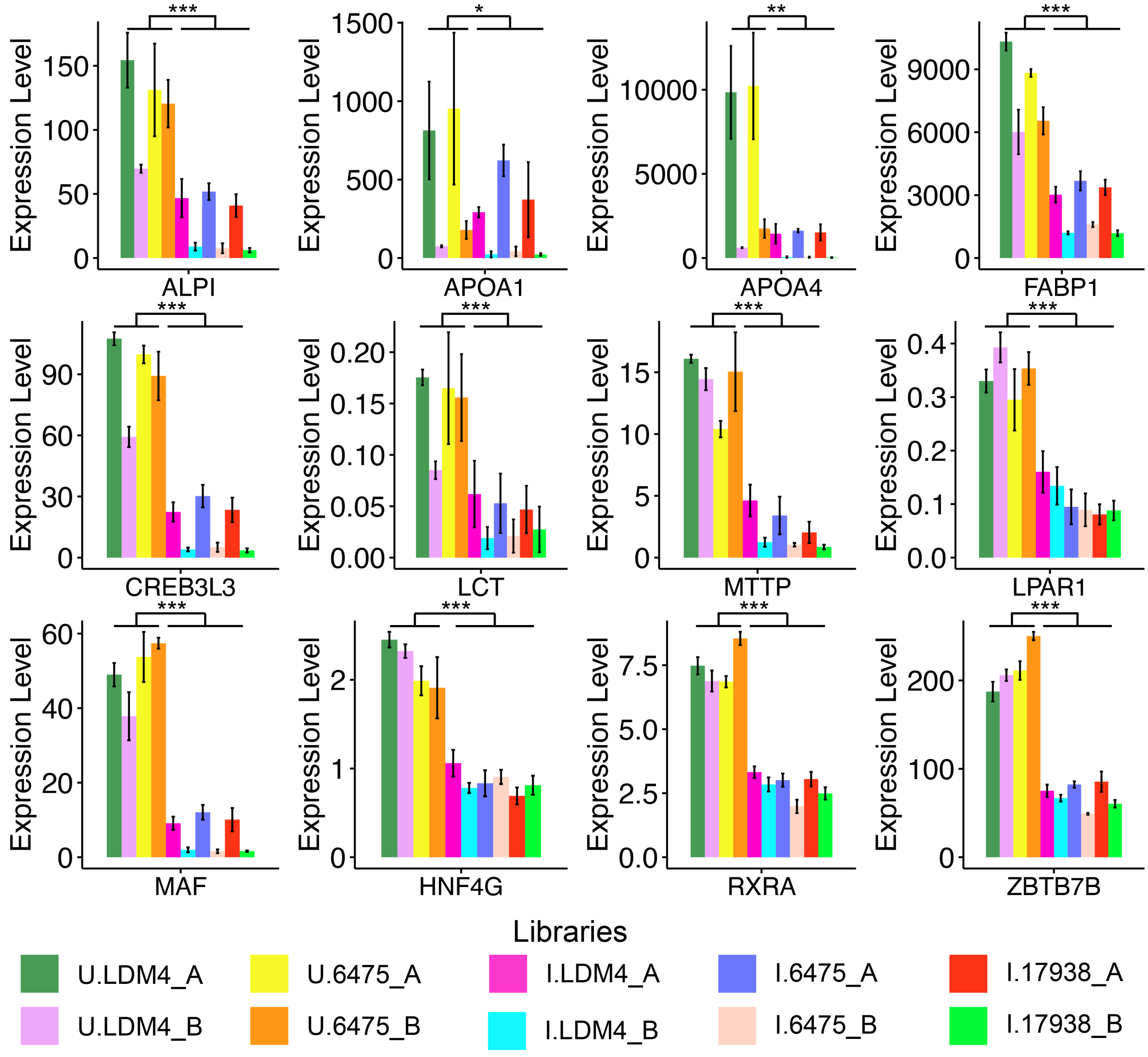

Supplemental Figure 1. Expression levels of enterocyte cell markers (*ALPI*, *APOA1*, *APOA4*, *FABP1*, *CREB3L3*, *LCT*, *MTTP*, *LPAR1*, *MAF*, *HNF4G*, *RXRA*, *ZBTB7B*)[1] in uninduced and induced NGN3-HIOs. Libraries are labeled "U" for uninduced, "I" for induced, and "A" and "B" for the first and second batches of *NGN3*-HIOs. Expression levels shown are counts per million GeTMM transformed read counts. Significance of expression levels between the uninduced and induced libraries was calculated using a two-sample, two-sided, Mann-Whitney test. *, $p<0.05$, **, $p<0.005$, ***, $p<0.0005$.

Supplemental Figure 2

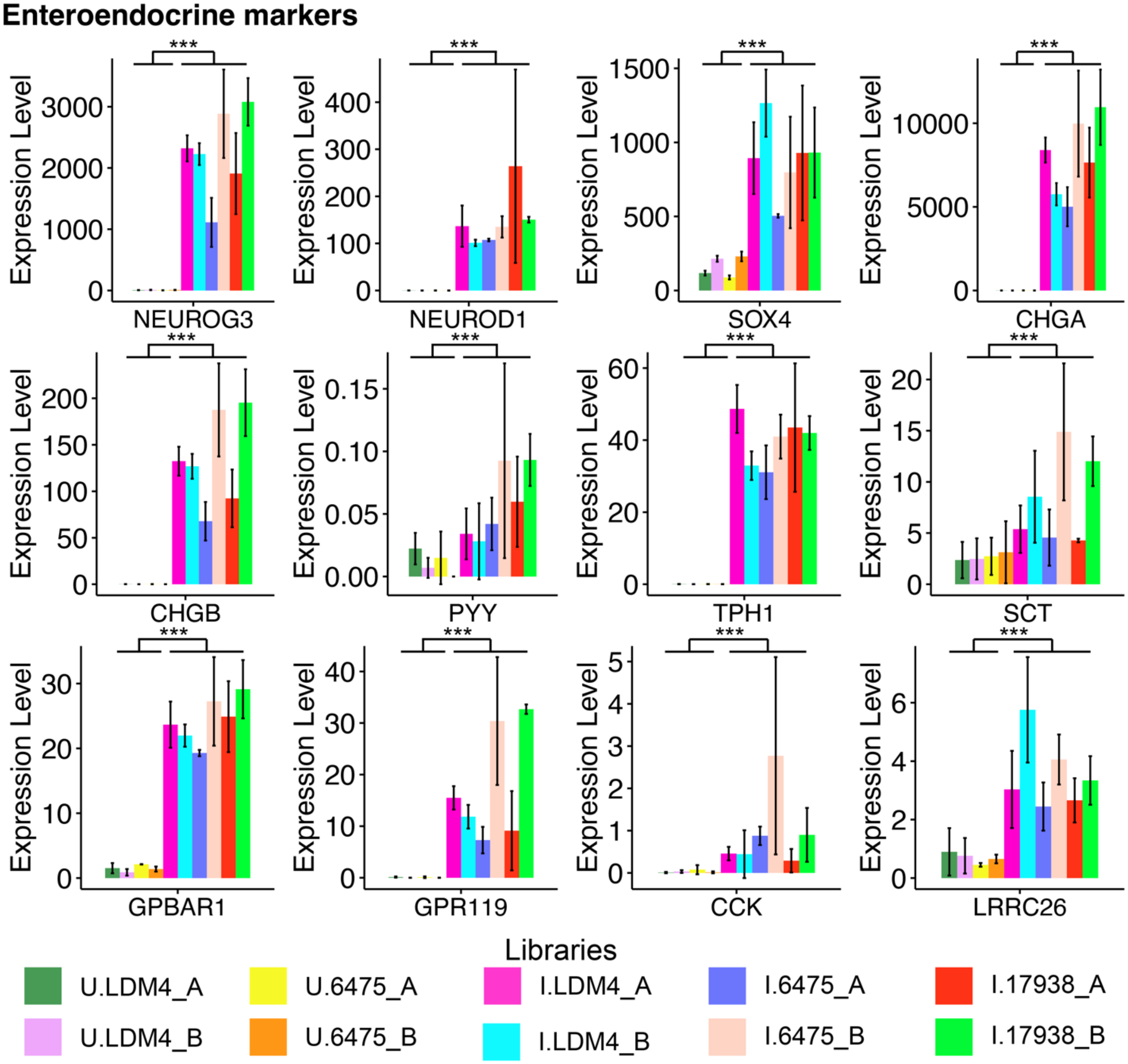

Supplemental Figure 2. Expression levels of enteroendocrine cell precursor markers (*NEUROG3*, *NEUROD1*, *SOX4*) and cell markers (*CHGA*, *CHGB*, *PYY*, *TPH1*, *SCT*, *GPBAR1*, *GPR119*, *CCK*, *LRRC26*) in uninduced and induced *NGN3*-HIOs. Libraries are labeled "U" for uninduced, "I" for induced, and "A" and "B" for the first and second batches of *NGN3*-HIOs. Expression levels shown are counts per million GeTMM transformed read counts. Significance of expression levels between the uninduced and induced libraries was calculated using a two-sample, two-sided, Mann-Whitney test. *, $p<0.05$, **, $p<0.005$, ***, $p<0.0005$.

Supplemental Figure 3

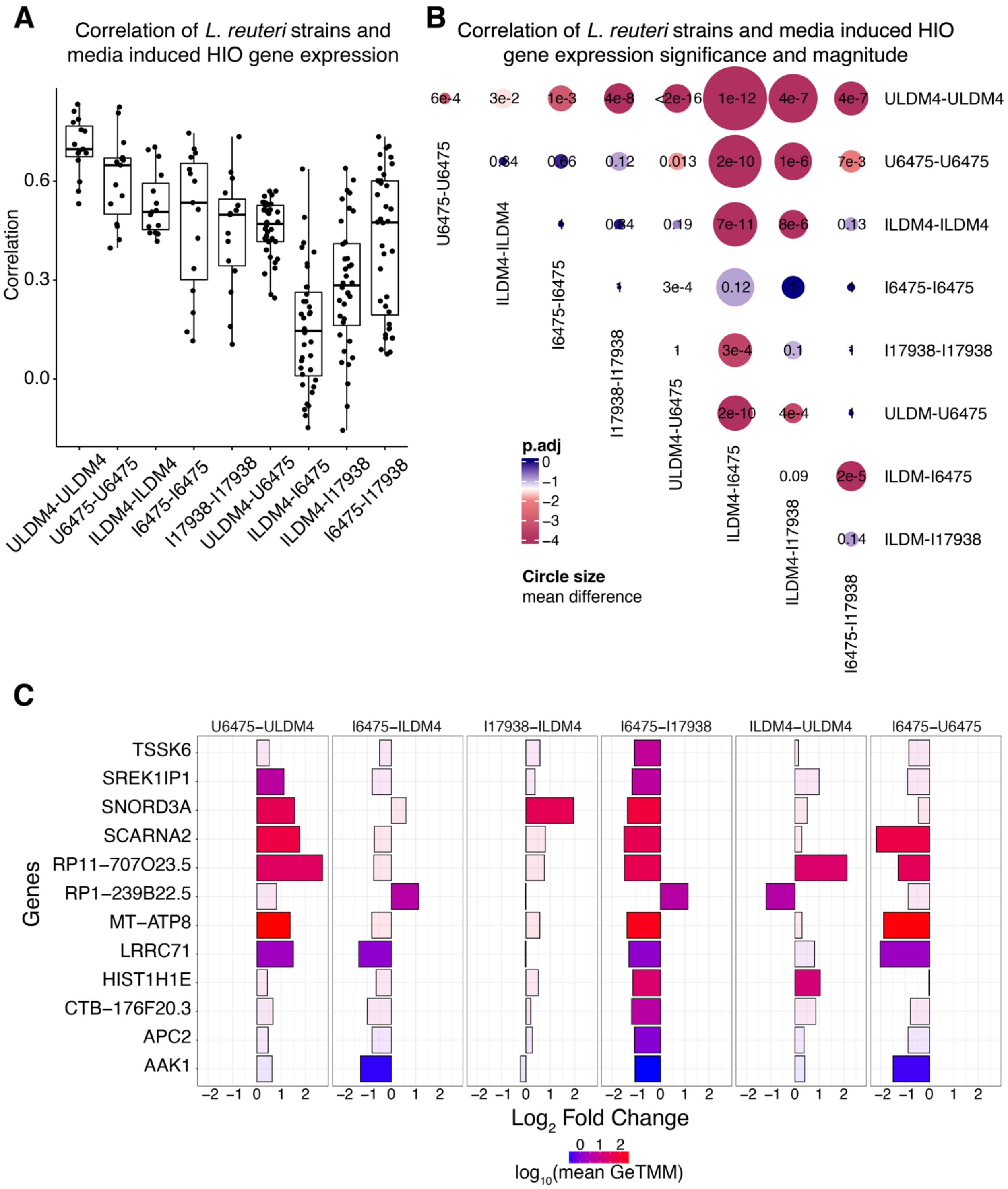

Supplemental Figure 3. Similarities and differences among *NGN3*-HIO transcriptomes. **A)** Boxplots of Pearson correlation values within and between transcriptomes. **B)**

Correlogram of mean differences from A (circle size) and adjusted p-values (circle fill and text in circle) between comparisons in A. **C)** Genes differentially regulated between *L. reuteri* 6475 and 17938 on induced *NGN3*-HIOs. The graph shows the $\log_2$ fold change expression of the gene for the indicated comparison. The bars are colored using the $\log_{10}$ scaled mean GeTMM counts to illustrate how abundantly expressed the gene is. Transparent overlays are used on genes not differentially expressed for the given comparison. Comparisons shown: U6475-ULDM4, *L. reuteri* 6475 on uninduced HIOs compared to LDM4 media control; I6475-ILDM4, *L. reuteri* 6475 on induced HIOs compared to LDM4 media control; I17938-ILDM4, *L. reuteri* 17938 on induced HIOs compared to LDM4 media control; I6475-I17938 *L. reuteri* 6475 compared to *L. reuteri* 17938 on induced HIOs; ILDM4-ULDM4, LDM4 media control on induced versus uninduced HIOs; I6475-U6475, *L. reuteri* 6475 on induced versus uninduced HIOs. For each, positive fold changes indicate genes upregulated by the condition listed first.

Supplemental Figure 4

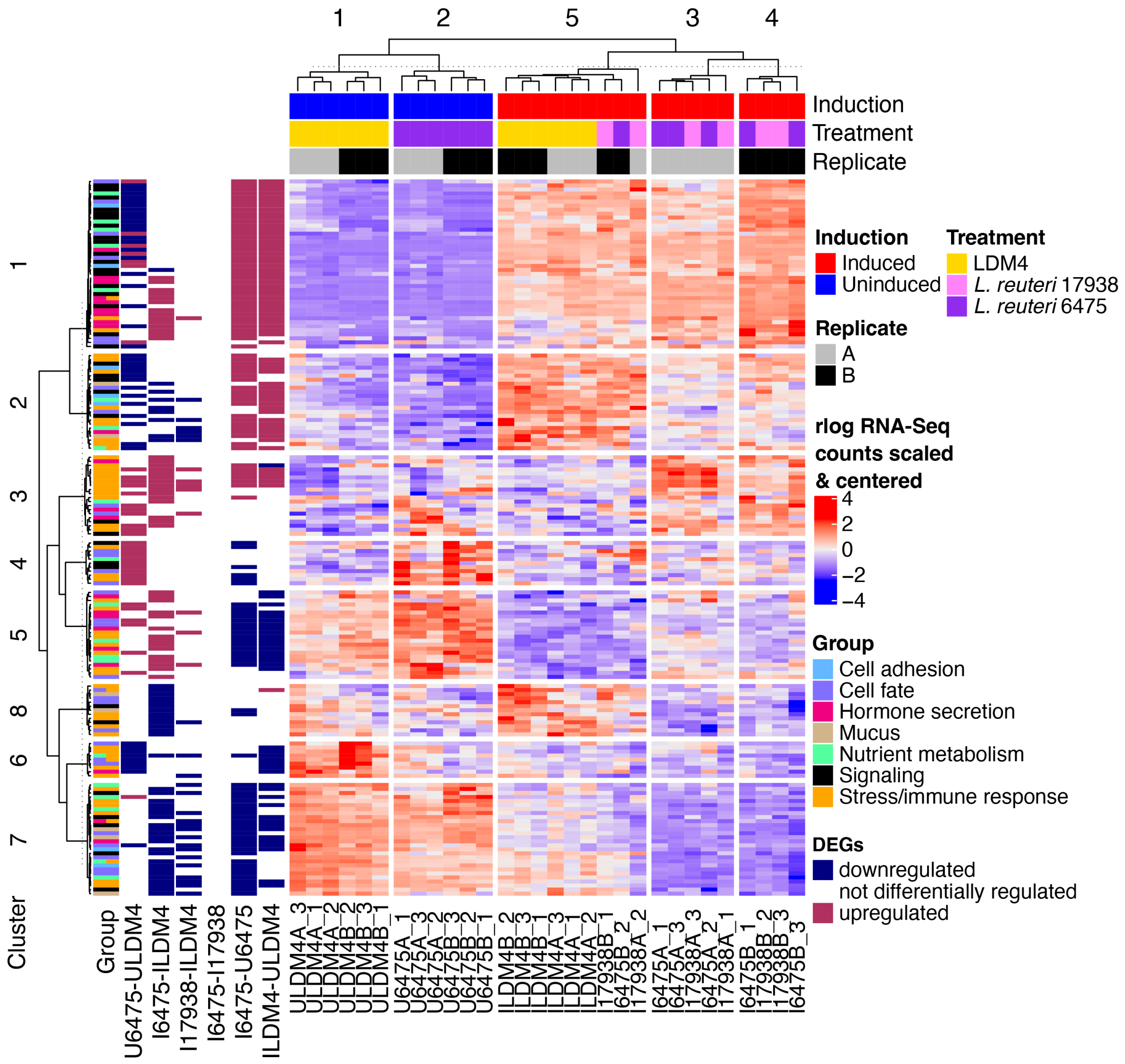

Supplemental Figure 4. Cluster analysis of DEGs belonging to functionally enriched groups. The heatmap shows gene expression values as rlog counts that were scaled and centered. Samples (the columns) along the bottom of the heatmap are labeled as “U” for uninduced *NGN3*-HIOs, “I” for induced *NGN3*-HIOs, “LDM4” for media only treatment (LDM4 media), “6475” for *L. reuteri* 6475 treatment, “17938” for *L. reuteri* 17938 treatment, “A” or “B” for the biological replicate, and “1”, “2”, “3” for the technical replicate within each biological replicate. Samples are annotated above the heatmap. Genes (rows) were arranged by K-means clustering and annotated into groups. For each sample comparison (e.g., U6475-ULDM4), if the gene was down or upregulated (e.g., higher in U6475 than ULDM4), a color is given.

Supplemental Figure 5

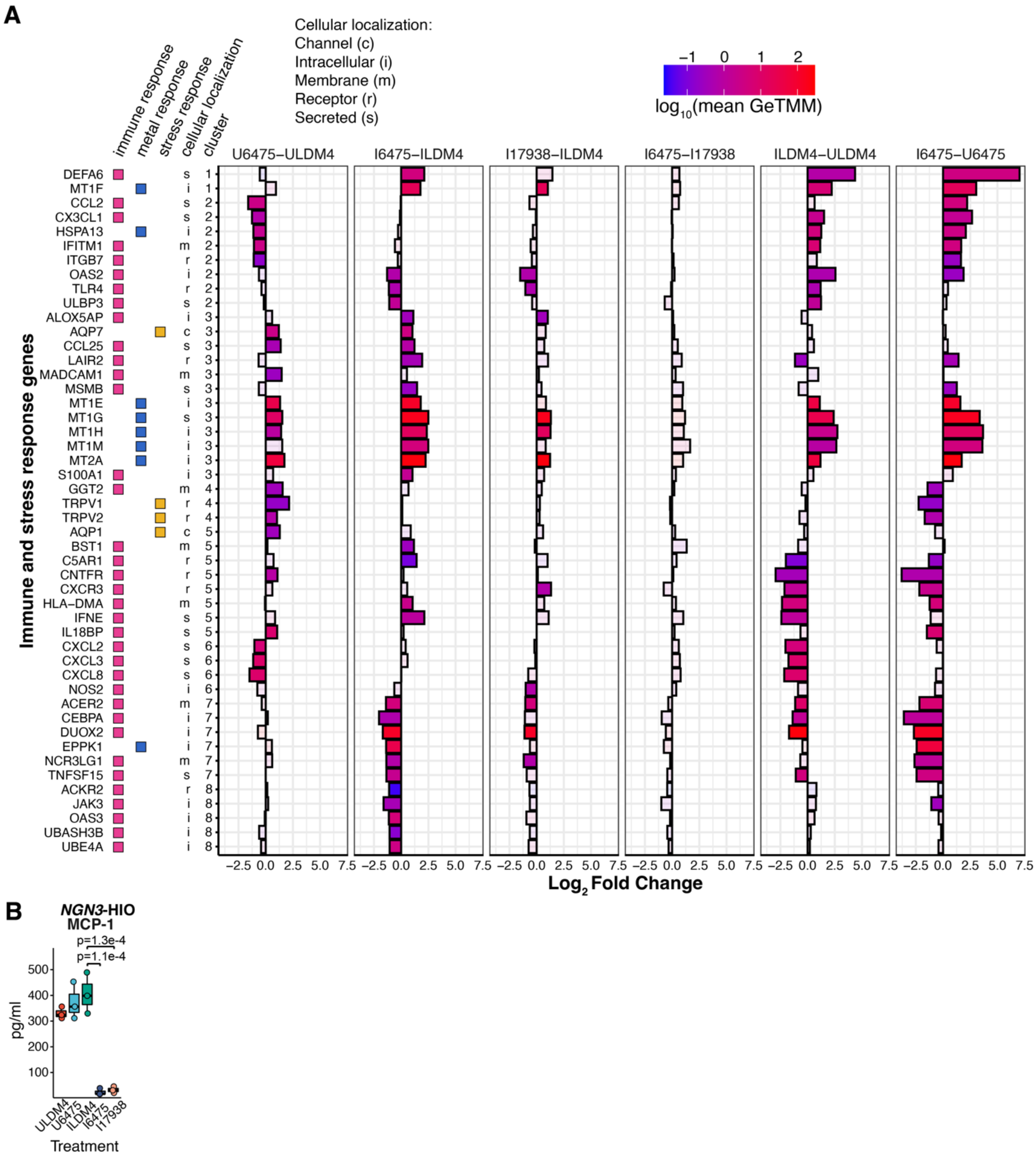

Supplemental Figure 5. *L. reuteri* regulates immune, metal, and stress response. **A)** Immune, metal, and stress genes differentially regulated by *L. reuteri*. The genes are annotated with their function, their cellular localization/type (channel (c), intracellular (i), membrane (m), receptor (r), secreted (s)), and what cluster they belong to relative to Supplemental Figure 4. The graph shows the $\log_2$ fold change expression of the gene for the indicated comparison. The bars are colored using the $\log_{10}$ scaled mean GeTMM counts to illustrate how abundantly expressed the gene is. Transparent overlays are

used on genes not differentially expressed for the given comparison. Comparisons shown: U6475-ULDM4, *L. reuteri* 6475 on uninduced HIOs compared to LDM4 media control; I6475-ILDM4, *L. reuteri* 6475 on induced HIOs compared to LDM4 media control; I17938-ILDM4, *L. reuteri* 17938 on induced HIOs compared to LDM4 media control; I6475-I17938 *L. reuteri* 6475 compared to *L. reuteri* 17938 on induced HIOs; ILDM4-ULDM4, LDM4 media control on induced versus uninduced HIOs; I6475-U6475, *L. reuteri* 6475 on induced versus uninduced HIOs. For each, positive fold changes indicate genes upregulated by the condition listed first. **B)** MCP-1 protein levels measured by Luminex on uninduced (U) or induced (I) HIOs treated with *L. reuteri* 6475 or 17938. Significance was determined with a Dunnett's Test, with $p<0.05$ being considered significant. Only p-values <0.05 are shown.

Supplemental Figure 6

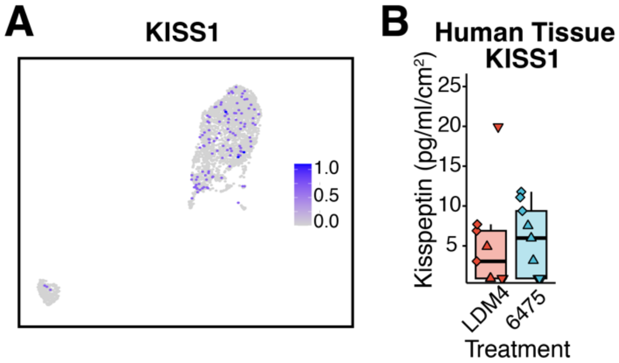

Supplemental Figure 6: KISS1 may be produced in the intestinal epithelium. **A)** UMAP of KISS1 using the Gut Cell Atlas adult jejunum data. **B)** Lack of kisspeptin secretion in response to bacterial media control (LDM4) and *L. reuteri* 6475 conditioned media from *ex vivo* human jejunal intestinal tissue. Shape represents unique human intestinal donors. Significance was determined using a linear mixed model with $p < 0.05$ considered significant. Only p-values < 0.05 are shown.

Supplemental Figure 7

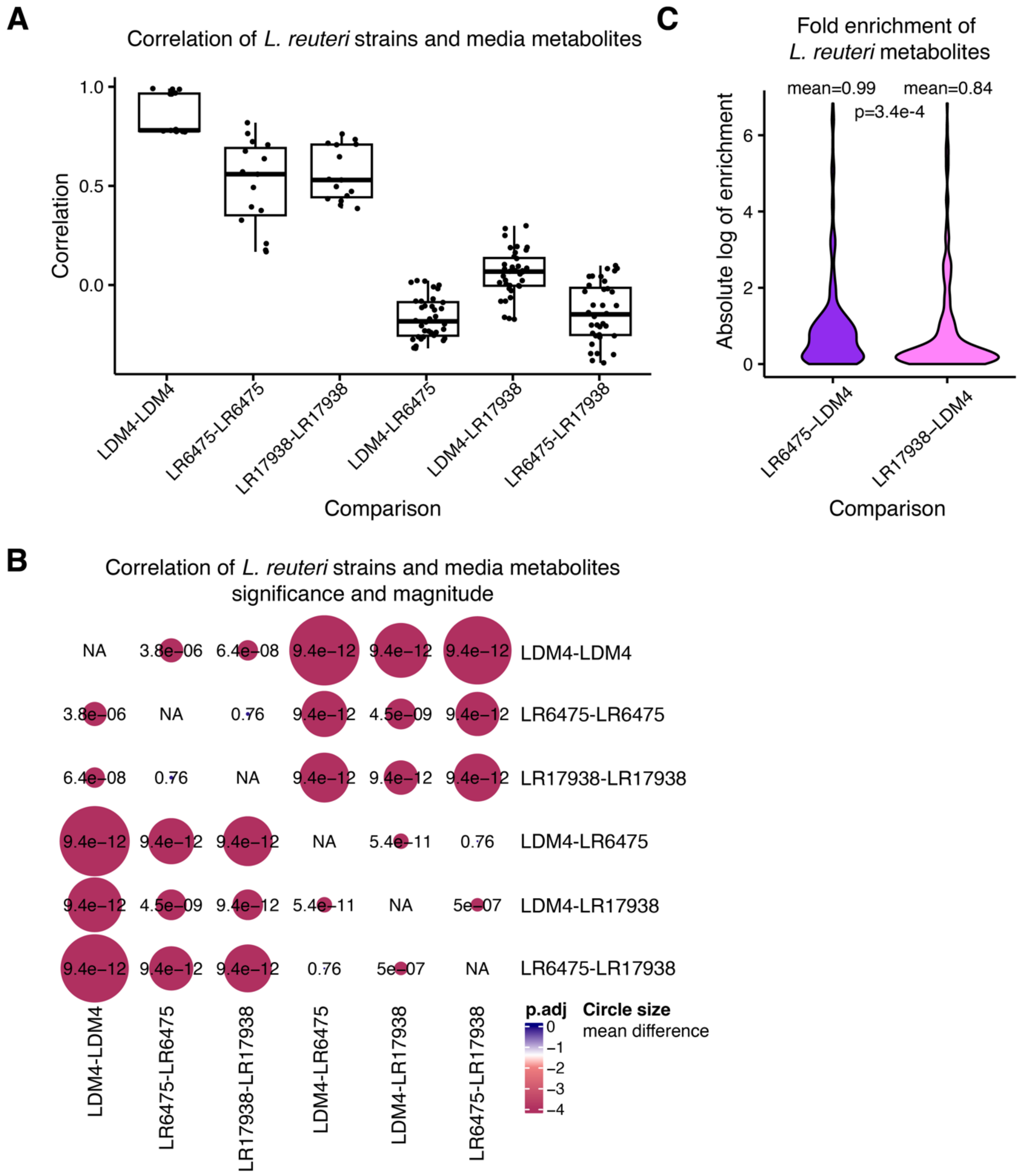

Supplementary Figure 7: *L. reuteri* 6475 and 17938 conditioned media metabolites. **A)** Spearman correlation of *L. reuteri* conditioned media metabolites and LDM4 media metabolites using normalized imputed data. **B)** Correlogram of mean differences (circle size) and adjusted p-values (circle fill and text in circle) from the comparisons shown in A. **C)** Absolute value of the log fold enrichment of *L. reuteri* conditioned media

metabolites compared to LDM4 media using normalized imputed data. The p-value was determined by a Wilcoxon test.

Supplemental Table 1: Sequencing reads per library. Number of sequencing reads for each sample after filtering and aligning to the reference human genomes (see Methods).

Supplemental Table 2: Genes differentially regulated between *L. reuteri* strains 6475 and 17938 in induced and uninduced HIOs. Libraries are labeled “U” for uninduced, “I” for induced, and “A” and “B” for the first and second batches of enteroids. For each comparison column, e.g. U6475-ULDM, “0” means no difference, “-1” means ULDM has higher expression values than U6475, and “1” means U6475 has higher expression values than ULDM. Expression levels shown are computed using the rlog. Output from DESeq2 (base mean (average of count values post normalization for size factors), $\log_2$ fold change, $\log_2$ fold change standard error, test statistic from a Wald test, p-value, and adjusted p-value using the Benjamini-Hochberg procedure) for each comparison are given.

Supplemental Table 3: Enriched functional groups in *L. reuteri* over media alone DEGs. Annotations were taken from the indicated annotation group as annotated by the PANTHER classification system and Reactome annotated pathways. Groupings were manually assigned to generalize the types of functional groups among the data. Enriched refers to whether the functional group is enriched in the set of DEGs or depleted. DEG Upregulated (+) or Downregulated (-) displays if the genes within the functional group were up- or downregulated by the respective *L. reuteri* strain compared to the media alone control.

Supplemental Table 4: Functional DEGs in *L. reuteri* over media alone annotations. DEGs belonging to a functional group are annotated at three levels (upper, middle, and final) with increasing levels of resolution. As well, the DEGs are classified by a subtype, giving information about their cellular location. DEG up- or downregulation information, gene information, and output from DESeq2 are presented as in Supplemental Table 2. GeTMM transformed read counts are given as well.

Supplemental Table 5: Metabolites measured by metabolomics in *L. reuteri* 6475 and 17938 conditioned media and LDM4 media. “CHEM_ID” is the identifier used for the metabolites in the metabolomics data. *Metabolite identity not confirmed based on a standard.

Supplemental Table 6: Unnormalized metabolite peak areas for each metabolite. Metabolites are indicated by their CHEM_ID in the columns, and samples are shown in the rows where “A” or “B” refers to media batch preparation and “1”, “2”, or “3” refers to sample replicate.

Supplemental Table 7: Normalized metabolite data for each metabolite. Metabolites are indicated by their CHEM_ID in the columns, and samples are shown in the rows where “A” or “B” refers to media batch preparation and “1”, “2”, or “3” refers to sample replicate.

Supplemental Table 8: Normalized imputed metabolite data for each metabolite. Metabolites are indicated by their CHEM_ID in the columns, and samples are shown in the rows where “A” or “B” refers to media batch preparation and “1”, “2”, or “3” refers to sample replicate.

Supplemental Table 9: Means, fold changes, and significance values among *L. reuteri* conditioned media and control media. *Metabolite identity not confirmed based on a standard.

Supplemental Table 10: Means, fold changes, and significance values among *L. reuteri* conditioned media and control media of metabolites significantly different from LDM4 in *L. reuteri* 6475 only. *Metabolite identity not confirmed based on a standard.

Supplemental Table 11: Means, fold changes, and significance values among *L. reuteri* conditioned media and control media of metabolites significantly different from LDM4 in *L. reuteri* 17938 only.

Supplemental Table 12: Means, fold changes, and significance values among *L. reuteri* conditioned media and control media of metabolites significantly different from LDM4 for *L. reuteri* 6475 or 17938 and between *L. reuteri* 6475 and 17938. *Metabolite identity not confirmed based on a standard.

REFERENCES

1. Haber, A.L., Biton, M., Rogel, N., Herbst, R.H., Shekhar, K., Smillie, C., Burgin, G., Delorey, T.M., Howitt, M.R., Katz, Y., et al. (2017). A single-cell survey of the small intestinal epithelium. Nature *551*, 333–339. https://doi.org/10.1038/nature24489.
